# Screening and Rapid Molecular Diagnosis of Tuberculosis in Prisons in Russia and Eastern Europe: A Cost-Effectiveness Analysis

**DOI:** 10.1371/journal.pmed.1001348

**Published:** 2012-11-27

**Authors:** Daniel E. Winetsky, Diana M. Negoescu, Emilia H. DeMarchis, Olga Almukhamedova, Aizhan Dooronbekova, Dilshod Pulatov, Natalia Vezhnina, Douglas K. Owens, Jeremy D. Goldhaber-Fiebert

**Affiliations:** 1 Stanford University School of Medicine, Stanford, California, United States of America; 2 Department of Management Science and Engineering, Stanford University, Stanford, California, United States of America; 3 AIDS Foundation East-West, Amsterdam, The Netherlands; 4 Veterans Affairs Palo Alto Health Care System, Palo Alto, California, United States of America; 5 Center for Health Policy/Center for Primary Care and Outcomes Research, Stanford University, Stanford, California, United States of America; World Health Organization, United States of America

## Abstract

**Background:**

Prisons of the former Soviet Union (FSU) have high rates of multidrug-resistant tuberculosis (MDR-TB) and are thought to drive general population tuberculosis (TB) epidemics. Effective prison case detection, though employing more expensive technologies, may reduce long-term treatment costs and slow MDR-TB transmission.

**Methods and Findings:**

We developed a dynamic transmission model of TB and drug resistance matched to the epidemiology and costs in FSU prisons. We evaluated eight strategies for TB screening and diagnosis involving, alone or in combination, self-referral, symptom screening, mass miniature radiography (MMR), and sputum PCR with probes for rifampin resistance (Xpert MTB/RIF). Over a 10-y horizon, we projected costs, quality-adjusted life years (QALYs), and TB and MDR-TB prevalence. Using sputum PCR as an annual primary screening tool among the general prison population most effectively reduced overall TB prevalence (from 2.78% to 2.31%) and MDR-TB prevalence (from 0.74% to 0.63%), and cost US$543/QALY for additional QALYs gained compared to MMR screening with sputum PCR reserved for rapid detection of MDR-TB. Adding sputum PCR to the currently used strategy of annual MMR screening was cost-saving over 10 y compared to MMR screening alone, but produced only a modest reduction in MDR-TB prevalence (from 0.74% to 0.69%) and had minimal effect on overall TB prevalence (from 2.78% to 2.74%). Strategies based on symptom screening alone were less effective and more expensive than MMR-based strategies. Study limitations included scarce primary TB time-series data in FSU prisons and uncertainties regarding screening test characteristics.

**Conclusions:**

In prisons of the FSU, annual screening of the general inmate population with sputum PCR most effectively reduces TB and MDR-TB prevalence, doing so cost-effectively. If this approach is not feasible, the current strategy of annual MMR is both more effective and less expensive than strategies using self-referral or symptom screening alone, and the addition of sputum PCR for rapid MDR-TB detection may be cost-saving over time.

*Please see later in the article for the Editors' Summary*

## Abstract 2

Daniel Winetsky and colleagues investigate eight strategies for screening and diagnosis of tuberculosis within prisons of the former Soviet Union.

## Editors' Summary

**Background:**

Tuberculosis (TB)—a contagious bacterial disease—is a major public health problem, particularly in low- and middle-income countries. In 2010, about nine million people developed TB, and about 1.5 million people died from the disease. *Mycobacterium tuberculosis*, the bacterium that causes TB, is spread in airborne droplets when people with active disease cough or sneeze. The characteristic symptoms of TB include fever, a persistent cough, and night sweats. Diagnostic tests include sputum smear microscopy (examination of mucus from the lungs for *M. tuberculosis* bacilli), mycobacterial culture (growth of *M. tuberculosis* from sputum), and chest X-rays. TB can also be diagnosed by looking for fragments of the *M. tuberculosis* genetic blueprint in sputum samples (sputum PCR). Importantly, sputum PCR can detect the genetic changes that make *M. tuberculosis* resistant to rifampicin, a constituent of the cocktail of antibiotics that is used to cure TB. Rifampicin resistance is an indicator of multidrug-resistant TB (MDR-TB), the emergence of which is thwarting ongoing global efforts to control TB.

**Why Was This Study Done?:**

Prisons present unique challenges for TB control. Overcrowding, poor ventilation, and inadequate medical care increase the spread of TB among prisoners, who often come from disadvantaged populations where the prevalence of TB (the proportion of the population with TB) is already high. Prisons also act as reservoirs for TB, recycling the disease back into the civilian population. The prisons of the former Soviet Union, for example, which have extremely high rates of MDR-TB, are thought to drive TB epidemics in the general population. Because effective identification of active TB among prison inmates has the potential to improve TB control outside prisons, the World Health Organization recommends active TB case finding among prisoners using self-referral, screening with symptom questionnaires, or screening with chest X-rays or mass miniature radiography (MMR). But which of these strategies will reduce the prevalence of TB in prisons most effectively, and which is most cost-effective? Here, the researchers evaluate the relative effectiveness and cost-effectiveness of alternative strategies for screening and diagnosis of TB in prisons by modeling TB and MDR-TB epidemics in prisons of the former Soviet Union.

**What Did the Researchers Do and Find?:**

The researchers used a dynamic transmission model of TB that simulates the movement of individuals in prisons in the former Soviet Union through different stages of TB infection to estimate the costs, quality-adjusted life years (QALYs; a measure of disease burden that includes both the quantity and quality of life) saved, and TB and MDR-TB prevalence for eight TB screening/diagnostic strategies over a ten-year period. Compared to annual MMR alone (the current strategy), annual screening with sputum PCR produced the greatest reduction in the prevalence of TB and of MDR-TB among the prison population. Adding sputum PCR for detection of MDR-TB to annual MMR screening did not affect the overall TB prevalence but slightly reduced the MDR-TB prevalence and saved nearly US$2,000 over ten years per model prison of 1,000 inmates, compared to MMR screening alone. Annual sputum PCR was the most cost-effective strategy, costing US$543/QALY for additional QALYs gained compared to MMR screening plus sputum PCR for MDR-TB detection. Other strategies tested, including symptom screening alone or combined with sputum PCR, were either more expensive and less effective or less cost-effective than these two options.

**What Do These Findings Mean?:**

These findings suggest that, in prisons in the former Soviet Union, annual screening with sputum PCR will most effectively reduce TB and MDR-TB prevalence and will be cost-effective. That is, the cost per QALY saved of this strategy is less than the per-capita gross domestic product of any of the former Soviet Union countries. The paucity of primary data on some facets of TB epidemiology in prisons in the former Soviet Union and the assumptions built into the mathematical model limit the accuracy of these findings. Moreover, because most of the benefits of sputum PCR screening come from treating the MDR-TB cases that are detected using this screening approach, these findings cannot be generalized to prison settings without a functioning MDR-TB treatment program or with a very low MDR-TB prevalence. Despite these and other limitations, these findings provide valuable information about the screening strategies that are most likely to interrupt the TB cycle in prisons, thereby saving resources and averting preventable deaths both inside and outside prisons.

**Additional Information:**

Please access these websites via the online version of this summary at http://dx.doi.org/10.1371/journal.pmed.1001348.

- The World Health Organization provides information (in several languages) on all aspects of tuberculosis, including general information on tuberculosis diagnostics and on tuberculosis in prisons; a report published in the *Bulletin of the World Health Organization* in 2006 describes tough measures taken in Russian prisons to slow the spread of TB
- The Stop TB Partnership is working towards tuberculosis elimination; patient stories about tuberculosis are available (in English and Spanish)
- The US Centers for Disease Control and Prevention has information about tuberculosis, about its diagnosis, and about tuberculosis in prisons (some information in English and Spanish)
- A PLOS Medicine Research Article by Iacapo Baussano et al. describes a systematic review of tuberculosis incidence in prisons; a linked editorial entitled The Health Crisis of Tuberculosis in Prisons Extends beyond the Prison Walls is also available
- The Tuberculosis Survival Project, which aims to raise awareness of tuberculosis and provide support for people with tuberculosis, provides personal stories about treatment for tuberculosis; the Tuberculosis Vaccine Initiative also provides personal stories about dealing with tuberculosis
- MedlinePlus has links to further information about tuberculosis (in English and Spanish)

## Introduction

Despite various control efforts, tuberculosis (TB) remains a major public health challenge in much of the developing and transitioning world, with an estimated 9.4 million new cases and nearly 2 million deaths in 2009 [1]. Expenditures for TB control efforts were expected to reach US$5 billion by 2011 [1]. The emergence of multidrug-resistant TB (MDR-TB) threatens to overwhelm recent gains in disease control and substantially increase costs, given that it requires lengthy and expensive treatment regimens [1].

Prisons present unique challenges for TB control because of malnutrition, overcrowding, and prolonged exposures. Furthermore, as prisoners are released and reenter the general population, prison TB epidemics have profound implications for general population health [2]. In Eastern Europe and Central Asia, where the prevalence of MDR-TB is among the highest worldwide, increased rates of incarceration are associated with increased civilian rates of MDR-TB and account for up to 60% of increased TB incidence in the general population [3].

Ongoing identification of active TB among inmates—including self-referral, screening, and bacteriological diagnosis of disease—is an important component of infection control in prisons, as it allows for early isolation and treatment of infectious cases that might otherwise go undetected [4],[5]. In prisons in which TB is highly endemic, such as those of the former Soviet Union (FSU), the World Health Organization (WHO) recommends ongoing active case finding for TB, yet does not outline a specific preferred methodology. The WHO recommends that all inmates be screened with chest X-ray upon entry to prison, and proposes three alternative methods for TB identification among the incarcerated population: self-referral (no screening), screening with symptom questionnaires, and radiographic screening with chest X-ray or mass miniature radiography (MMR) [4],[5]. According to these recommendations, cases suspected on the basis of screening should be followed up with bacteriologic diagnosis by direct sputum smear microscopy and, if resources allow, with sputum culture.

Administrators of most prisons in FSU countries rely on annual radiographic screening with MMR to find TB cases within their incarcerated populations. MMR involves taking a 7×7 cm photofluorographic image of the entire thoracic cavity and examining it with the aid of a magnified light box. With the use of a mobile photofluorography machine, hundreds of inmates can be screened in a matter of days. However, the sensitivity of MMR for active pulmonary TB has not been well studied and may be low compared with conventional chest X-rays [6]. Cases of active TB detected on screening are diagnosed bacteriologically with direct sputum smear microscopy and in some cases sputum culture.

Previous modeling studies have suggested that annual radiographic screening of inmates can lower prevalence over time, compared with passive detection alone [7]. However, radiographic imaging is neither sensitive nor specific for active pulmonary TB [8], and it is unclear what the optimal combination of methods is for settings with significant resource constraints and a high burden of MDR-TB requiring large outlays for treatment. Furthermore, new technologies such as sputum PCR are available for rapid molecular diagnosis of infection with *Mycobacterium tuberculosis*, but it is unclear if these technologies are cost-effective for use in highly endemic prisons.

In this study, we modeled simultaneous TB and MDR-TB epidemics in prisons of the FSU to evaluate the relative effectiveness and cost-effectiveness of alternative strategies for screening and diagnosis of active TB available to prison health administrators where a significant proportion of TB cases are MDR-TB. We developed models to simulate TB epidemics in an average prison in the FSU, as well as prisons in three individual republics with distinct epidemic dynamics and per-capita income levels.

## Methods

### Model Structure

**Figure 1:**
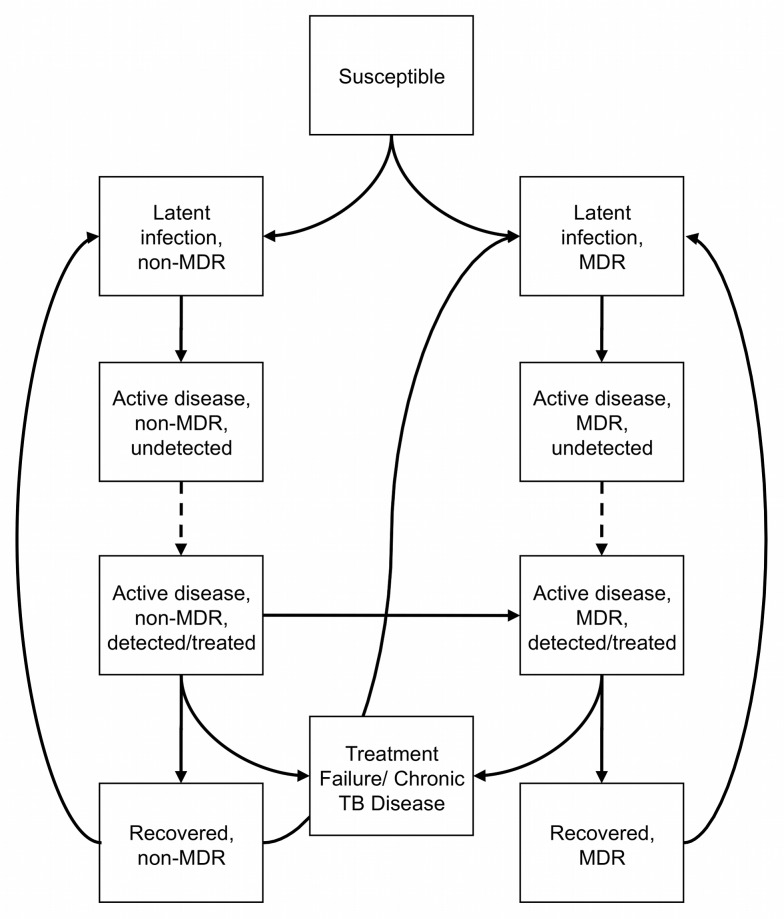
Natural history, diagnosis, and treatment of TB. Simplified diagram of the health states and transitions in our model. Screening and diagnostic alternatives affect the rate of transition from undetected to detected active disease, represented here by a dashed arrow. Death from all states is not shown. See Figures S1 and S2 for more detail regarding the model structure.

We developed a dynamic transmission model of TB and MDR-TB epidemics in prisons of the FSU each housing 1,000 inmates—roughly the average prison size in this region. Each individual prisoner can transition between health states that represent the natural history of infection with *M. tuberculosis* (Figure 1). Prisoners enter the population through arrest and sentencing, at which point they are screened once with chest X-ray; prisoners leave the population through release, death from active TB disease, or death from other causes. Exit and entry rates are held equal so that the size of the population remains fixed at 1,000. Our model is a deterministic, population-based compartmental model, with transitions between health states modeled by a set of ordinary differential equations (Table S5). Based on an exponential assumption, we applied an exponential transform to convert probabilities into time-constant rates. Our set of differential equations was integrated using Matlab 7.12 (MathWorks) using a 1-wk time step. Total quality-adjusted life years (QALYs) and costs accrued in the transmission model and in cohort Markov chains calculating remaining life years after release (see below) were calculated for use in a static decision analytic framework (Figure S3). No time-series data could be identified with which to calibrate our model, but the prevalence of latent infection in the general population was varied manually within observed ranges to achieve a match to the observed prevalence of active disease within prisons (Text S1).

### Natural History

Susceptible individuals can become infected with TB that is either multidrug-resistant (MDR-TB) or sensitive to isoniazid and/or rifampin (non-MDR-TB) (Figure 1), and develop either a slow- or fast-progressing latent infection. Latently infected individuals remain noninfectious until they progress to active disease. Active disease can be either smear-positive or smear-negative, which convey different levels of infectivity.

Individuals with active disease remain infectious until they are detected, isolated, and treated appropriately or until they die. The process of detection can occur either through self-referral or through screening, which is modeled as a discrete annual event. Screening and diagnostic strategies can reduce overall TB and MDR-TB prevalence by diminishing the pool of infectious cases.

**Figure 2:**
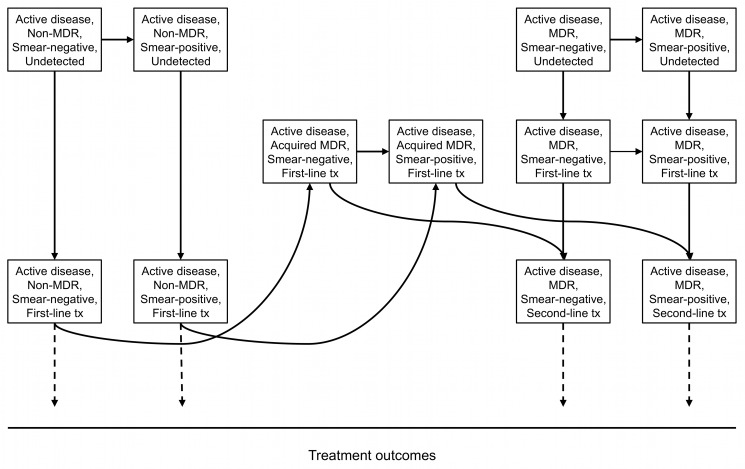
Diagnosis of non-MDR-TB and MDR-TB and the development of acquired (treatment-associated) MDR-TB. Diagram showing the detection of non-MDR-TB and MDR-TB. Individuals with MDR-TB transition through a state in which they are treated with standard first-line (DOTS) therapy before the multidrug resistance of their strain is detected. This rate of transition is substantially accelerated in strategies using sputum PCR (Xpert MTB/RIF). Individuals developing acquired (treatment-associated) MDR-TB also transition through a state in which they are treated with standard first-line treatment before the multidrug resistance of their strain is detected. The rate of detection for these individuals is unaffected by the choice of case finding strategy. tx, treatment.

Cases of non-MDR-TB are treated for 6 mo with first-line therapy, a standard four-drug regimen in accordance with the WHO's Directly Observed Therapy Short-Course (DOTS) strategy, and MDR-TB cases are treated with second-line therapy for 24 inpatient months with ≥5 drugs, in accordance with DOTS-Plus [9]. Treatment with second-line therapy is initiated on the basis of drug susceptibility testing, failure to improve or convert to sputum negativity after a second intensive phase of treatment with DOTS (category II), or—in strategies using rapid sputum PCR—detection of DNA specific to *M. tuberculosis* and rifampicin resistance with Xpert MTB/RIF (Figure 2). Individuals with MDR-TB being treated with standard first-line treatment remain infectious. Such individuals were assumed to have contact with the general prison population if smear-negative, and only with other smear-positive individuals being treated with standard first-line treatment if smear-positive. After successful treatment, individuals enter strain-specific recovered states with increased risk of relapse/reinfection. In the event of smear positivity at the end of a full first-line treatment course, individuals are re-treated. Individuals failing a second first-line treatment course or one 24-mo second-line treatment course enter a chronic disease state. Inadequate treatment and/or poor adherence in our model can lead to acquired MDR-TB as well as reinfection from those with MDR-TB strains who have yet to be placed on second-line treatment (Text S1). HIV infection was not explicitly considered (Text S1).

### Alternative Case Detection Strategies

We evaluated the cost-effectiveness of alternative strategies for screening with or without the use of rapid molecular diagnostic testing. We considered eight strategies for TB screening and diagnosis: no screening; MMR screening; symptom screening; sputum PCR screening; combined MMR and symptom screening; MMR screening with sputum PCR for rapid MDR-TB detection; symptom screening with sputum PCR for rapid MDR-TB detection; and combined MMR and symptom screening with sputum PCR for rapid MDR-TB detection. In the strategy of combined MMR and symptom screening, either a positive MMR result or positive symptom screen leads to further evaluation. Sputum PCR was used either as a stand alone screening test for active pulmonary TB, or in combination with other case finding strategies as a preliminary test for multidrug resistance among those found to have evidence of active TB on screening. Implicit in all strategies was the use of sputum culture and drug susceptibility testing in treatment planning for confirmed cases.

The sensitivity and specificity of each screening/diagnosis combination determines the proportion of individuals with undetected disease who are ultimately detected and treated (see Text S1 for estimates of sensitivity and specificity, and Table 1). We assumed conditional independence of tests used in combination, and this resulted in estimates comparable to available data. In strategies that include the use of sputum PCR, we assumed that individuals testing positive for rifampin resistance are started on second-line treatment within 1 wk. Hence, when sputum PCR is used in combination with other screening strategies, its use is able to impact MDR-TB transmission by reducing the opportunity for MDR-TB cases being treated with standard first-line treatment to infect other individuals while awaiting results from standard drug sensitivity testing and consequent transition to second-line treatment. Individuals falsely diagnosed with MDR-TB by sputum PCR in our model were given a full course of second-line treatment.

**Table 1:** Screening/diagnostic tools and their characteristics.

Diagnostic Tool	TB Form	Sensitivity		Specificity		References
		Value	Range	Value	Range	
**MMR**	Smear-negative	0.80	(0.79–0.82)	0.98	(0.980–0.982)	[37]–[39]
	Smear-positive	0.64	(0.59–0.69)			
**Symptom screening**	Smear-negative	0.30	(0.28–0.32)	0.89	(0.888–0.892)	[37]–[41]
	Smear-positive	0.58	(0.54–0.63)			
**Sputum PCR**	Smear-negative	0.68	(0.61–0.74)	0.99	(0.98–1.00)	[42]
	Smear-positive	0.98	(0.97–0.99)			
**Sputum PCR for rapid MDR-TB detection**	MDR	0.98	(0.95–1.00)	0.98	(0.97–0.99)	[42]

See Table S6 for further details on screening test characteristics.

### Costs and Outcomes

Taking the health system perspective, costs accrue from screening and diagnosis, treatment, and hospitalization, both while incarcerated and after release. All projected future costs are discounted at a 3% annual rate [10]. We estimated these costs for the FSU based on both primary data collection and data from published studies. We conducted a primary cost analysis of TB control methods in prisons of Tajikistan, the poorest FSU country, examining accounting data and conducting interviews with staff at local public health facilities and international nongovernmental organizations, using the ingredients-costing approach. We then developed aggregate unit cost estimates for conducting MMR, symptom screening, sputum smear microscopy (using Ziehl-Neelsen staining), sputum culture (using BACTEC MGIT in parallel with Lowenstein-Jensen medium), and drug susceptibility testing, and for non-MDR-TB and MDR-TB treatment (Table 2). These estimates took into account the costs of capital, supplies, labor, and administrative overhead [11]. All costs were converted to 2009 US dollars, and, where necessary, adjusted for inflation (Text S1). Fixed costs were depreciated linearly over their expected useful lifetime. To construct estimates for other FSU republics, we adjusted costs subject to significant local price variation using international data from the WHO and International Labour Organization [12],[13] (Text S1). We drew our estimate for the cost of sputum PCR analysis from an adaptation of the estimated ingredient costs reported in a recent large, multi-center in-trial cost-effectiveness analysis [14].

**Table 2:** Cost estimates.

Category	Cost of Method per Test or of Treatment per Case (Range)
**Screening/Diagnostic Method**	
MMR	$4.85 (3.64–6.06)
Symptom screening	$2.19 (1.64–2.74)
Sputum smear	$2.16 (1.62–2.70)
Sputum PCR	$24.08 (18.06–30.09)
**Treatment**	
Drug sensitive TB (smear-negative)	$364.45 (273.39–455.65)
Drug sensitive TB (smear-positive)	$441.42 (331.11–551.85)
Multi-drug resistant TB	$7,961.02 (5,970.90–9,951.50)

Costs given in US dollars. Values are from primary cost analysis except for sputum PCR, which was adjusted from [14]. Screening costs are applied to all individuals not currently being treated for active TB. Diagnostic costs are applied to those individuals who test positive and include only those additional tests and clinical evaluations not part of a given screening strategy's screening test. Further work-up costs to determine appropriate treatment (e.g., drug sensitivity testing) are included in treatment costs if they are not part of an earlier screening strategy. See Table S7 for further details.

Health states were adjusted for quality of life [15],[16]. We tracked the total number of QALYs lived by all inmates entering the prison over a 10-y time horizon, beginning at the time of entry into the prison and continuing until death (including life lived after release). Over the same time span, we tracked total costs spent by the health system. Expected healthcare costs and years of life lived after release were projected using simplified, age-stratified cohort Markov models based on the age distribution of Russian prisoners [17], age-based life-expectancy for the Russian Federation [18], average governmental health expenditures in FSU countries [19], and the probability of developing reactivation TB disease [20]. We did not model transmission occurring outside the prison system after release of inmates.

Our primary outcome was the number of incremental dollars spent for each additional QALY lived resulting from the choice of a particular strategy over the next best strategy (i.e., the incremental cost-effectiveness ratio [ICER]) [10]. Additional outcomes for each strategy included the following: total dollars spent, total QALYs gained, and the prevalence of TB (all forms) and MDR-TB.

### Demographic and Epidemiologic Data

Demographic and epidemiologic parameters were derived from the published literature (Table S1) [1],[17],[21]–[26]. In our main analysis, population-weighted averages of epidemiologic data from all FSU countries were used as inputs for the model. Under the current case detection strategy—MMR screening followed by smear microscopy—TB transmission was allowed to reach a steady-state equilibrium before this strategy was continued or other interventions were implemented.

Representing the spectrum of republics (from poorest to wealthiest), in country-specific situational analyses, the epidemiologic data of Tajikistan [18],[27] (AIDS Foundation East-West, unpublished data), the Russian Federation [17],[18],[24],[28], and Latvia [18],[29] were separately used as inputs for the model, and the remaining epidemiologic parameters were varied manually to match reported prevalence in TB prison surveys. Again, the models were allowed to reach steady-state equilibria for each republic before the assessment of alternative case finding strategies began.

For parameters related to treatment delivery and effectiveness, published outcomes of first-line (DOTS) and second-line (DOTS-Plus) treatment (which includes treatment of MDR-TB) programs in FSU prisons were used (Table 3). Outcomes of second-line treatment in prison settings were supplemented with available data from the civilian sector in former Soviet countries as well.

**Table 3:** Treatment outcomes in prisons in the FSU.

Outcome	Annualized Rate^a^	Range for Sensitivity Analyses	Approximate Proportion and Range^a^^,^ b	Model Parameter
**Non-MDR-TB ** [**23**],[**43**] a				
Treatment success^b^	1.78800	(0.73814–2.00248)	92.3% (78.2%–95.6%)	π_d_
Amplification of resistance^b^	0.08060	(0.05235–0.10981)	4.1% (2.4%–12.4%)	F
Treatment failure	0.05940	(0.03770–0.08121)	3.1% (1.7%–9.3%)	τ_d_
Death	0.00772	(0.00200–0.01511)	0.4% (0.1%–1.8%)	ζ_4d_
**MDR-TB ** [**9**],[29],[44],[**45**] a				
Treatment success	0.51400	(0.47467–0.55737)	71.4% (67.0%–75.8%)	π_m_
Treatment failure	0.16667	(0.14786–18626)	23.2% (19.7%–27.0%)	τ_m_
Death	0.03844	(0.02988–0.04716)	5.3% (3.9%–7.0%)	ζ_4m_

a See Text S1 for summary of literature review and methodology of estimation of rates and proportions of individuals with outcomes. Based on an exponential assumption, we applied an exponential transform to convert probabilities into time-constant rates. When more than one event could occur from a given health state, we computed the overall weekly likelihood of any event occurring based on the sum of the weekly rates relevant to that health state and then used the ratio of a particular rate to the sum of the relevant rates to determine the proportion of events of each type that occurred from that health state within the weekly interval.

b Acquisition of MDR-TB during first-line (DOTS) treatment (σ) can occur via amplification of existing resistance, which is static (F), or through reinfection, which is dynamic and depends on the local force of infection among those being treated with standard first-line therapy (not shown). The above computed proportions of individuals with outcomes are for illustrative purposes; they exclude cases of reinfection in their estimation and are based only on these static parameters. This implies that when MDR-TB transmission is higher, treatment success and treatment failure proportions for non-MDR-TB will be lower than shown in the table, since reinfection is a more substantial competing risk.

### Sensitivity and Uncertainty Analyses

In order to explore the impact of uncertainty around the input parameters on our findings, we performed a series of sensitivity analyses for our model. We conducted univariate sensitivity analyses, selected two-way sensitivity analyses, a probabilistic sensitivity analysis, and a number of situational analyses in which we tested alternative plausible scenarios. Where possible, parameter ranges for univariate and probabilistic sensitivity analyses were selected on the basis of 95% confidence intervals for individual parameter estimates (for pooled data, confidence intervals were adjusted for unobserved heterogeneity) (Text S1). Where confidence intervals could not be calculated, ranges were subjectively estimated. In univariate and two-way sensitivity analyses and situational analyses, the cost-effectiveness frontier was evaluated for changes in the number and order of strategies composing it. For the probabilistic sensitivity analysis, net monetary benefit (NMB) was calculated for randomly selected parameter sets drawn from the marginal distributions of each parameter, and the likelihood that each strategy was cost-effective was evaluated for varying willingness-to-pay thresholds. Probabilistic sensitivity analyses used 10,000 simultaneous samples from the uncertainty distributions of the model's inputs (i.e., second-order Monte Carlo sampling), propagating this joint uncertainty in the model parameters into the modeled outcomes, though because of the lack of appropriate data for empirical calibration, the distributions sampled generally assumed no correlation, unlike joint posterior distributions produced by empirical calibration procedures (Text S1).

### Ethics Statement

Our study was deemed exempt from review by the Stanford Human Subjects Review Committee because it utilizes only publicly available, de-identified data.

## Results

Of all case finding strategies considered, sputum PCR screening for TB in the general prison populations of the FSU results in the lowest prevalence rates of TB and MDR-TB and achieves these benefits at a cost per QALY that is well below the per-capita gross domestic product (GDP) of the FSU. If sputum PCR is not an available option as a primary screening tool for TB, annual MMR screening (either alone or in combination with symptom screening) is more effective and less costly than strategies that rely on symptom screening alone over the 10-y time horizon.

### Overall TB and MDR-TB Prevalence

**Figure 3:**
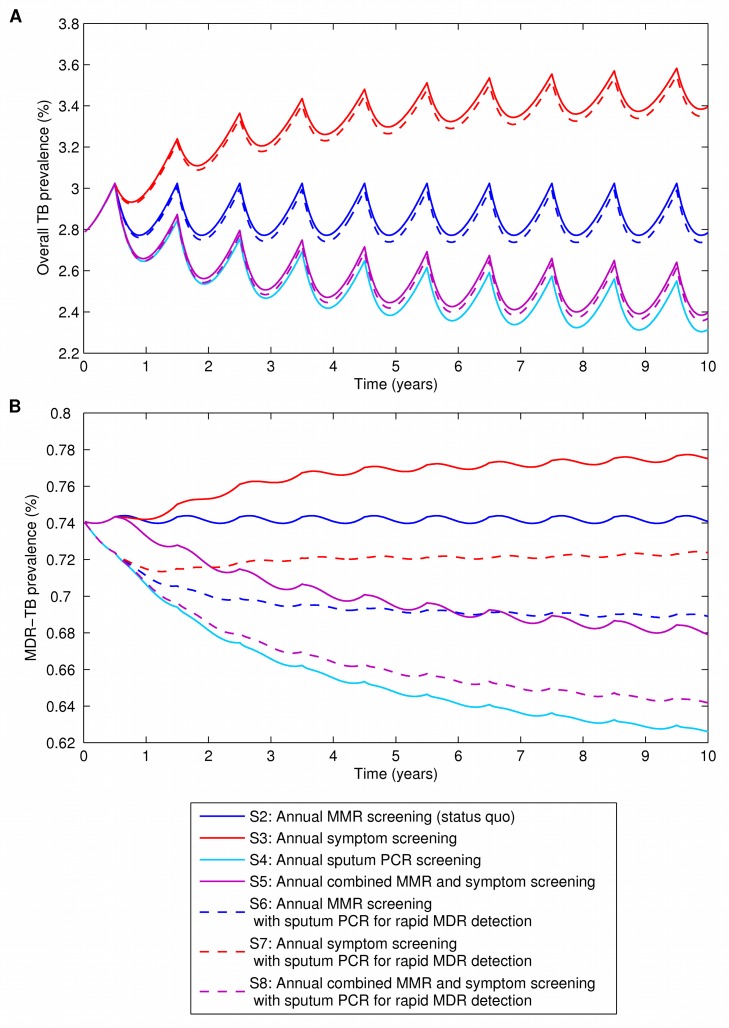
The effects of alternative screening and diagnostic strategies on TB and MDR-TB prevalence. (A) Prevalence of TB (both non-MDR-TB and MDR-TB) among prison population over 10-y time horizon. (B) Prevalence of MDR-TB among prison population over 10-y time horizon. Strategy 1 (S1), self-referral only (no screening), is not shown.

When used as a primary screening tool administered annually to the general prison population, sputum PCR led to the largest reduction in both overall TB prevalence and MDR-TB prevalence. Overall TB prevalence fell from 2.78% to 2.31%, and MDR-TB prevalence fell from 0.74% to 0.63% (Figure 3). This strategy also resulted in the largest number of QALYs gained.

Combining MMR and symptom screening into one annual activity reduced overall TB and MDR-TB prevalence substantially compared with MMR or symptom screening alone, though not as much as sputum PCR used as a primary screening tool. Compared with MMR screening alone, combining MMR and symptom screening reduced 10-y overall prevalence of TB to 2.40% and reduced MDR-TB prevalence to 0.68% (Figure 3). Similarly, combined MMR and symptom screening resulted in appreciable QALY gains (Table 4). Only the addition of sputum PCR or the use of sputum PCR by itself as a primary screening tool produced lower prevalence rates at 10 y.

**Table 4:** Costs, health effects, and ICERs for a prison of 1,000 individuals.

Strategy	Total Costs^a^	Total Health Benefits (QALYs)^a^	Prevalence of TB (Percent)^a^	Prevalence of MDR-TB (Percent)^a^	Strategy on Efficient Frontier	Incremental^b^ Costs^a^	Incremental^b^ Health Benefits (QALYs)^a^	ICER^b^ (Cost/QALY)^a^
MMR screening with sputum PCR detection of MDR-TB	$18,524,341 (14,987,150; 23,015,750)	79,886 (77,213; 82,093)	2.75 (1.69; 5.03)	0.69 (0.38; 1.88)	Reference^c^			
MMR screening (status quo)	$18,528,984 (14,988,050; 23,043,950)	79,869 (77,179; 82,073)	2.78 (1.71; 5.14)	0.74 (0.40; 2.05)	Dominated			
Combined MMR and symptom screening	$18,589,325 (15,037,000; 23,059,950)	79,971 (77,297; 82,164)	2.40 (1.52; 4.40)	0.68 (0.37; 1.86)	Extended Dominance^d^			
Sputum PCR screening	$18,595,892 (15,062,030; 23,042,880)	80,018 (77,381; 82,209)	2.31 (1.48; 4.21)	0.63 (0.35; 1.71)	Non-dominated	$71,551 (−56,000; 143,000)	132 (59; 312)	$543 (CS; 2,039)
Self-referral (no screening)	$18,604,958 (15,073,030; 23,155,950)	79,614 (76,828; 81,820)	4.28 (2.46; 8.29)	0.99 (0.51; 2.79)	Dominated			
Symptom screening	$18,608,052 (15,064,030; 23,106,970)	79,792 (77,087; 81,998)	3.39 (2.06; 6.31)	0.78 (0.41; 2.19)	Dominated			
Combined MMR and symptom screening with sputum PCR detection of MDR-TB	$18,627,632 (15,082,050; 23,082,970)	79,984 (77,334; 82,174)	2.37 (1.50; 4.33)	0.64 (0.36; 1.75)	Dominated			
Symptom screening with sputum PCR detection of MDR-TB	$18,633,929 (15,098,050; 23,129,550)	79,806 (77,120; 82,018)	3.36 (2.03; 6.23)	0.72 (0.39; 2.03)	Dominated			

All costs are given in 2009 US dollars. Quality of life weights used for these analyses are shown in Table S8. Shown are total health system costs accrued and total QALYs lived by individuals in the model over the 10-y time horizon, as well as overall TB prevalence and MDR-TB prevalence at the end of 10 y. For each non-dominated strategy, the additional cost for each QALY gained was evaluated in comparison to the next best strategy, giving the ICER. A strategy is considered “dominated” if there exists an alternative strategy that is both more effective and less costly or provides greater benefits more cost-effectively. In all scenarios, starting TB prevalence was 2.78% and MDR-TB prevalence was 0.74%.

a Numbers inside parentheses represent 95% confidence intervals based on the probabilistic sensitivity analysis results. While confidence intervals for many quantities are wide, there is correlation across screening strategies such that the confidence intervals around the differences between strategies are much smaller, and rank orderings of strategies are very frequently preserved. For example, sputum PCR screening produces the greatest health benefit >99.5% of the time, the greatest reduction in TB prevalence >99% of the time, and the greatest reduction in MDR-TB prevalence >99.5% of the time. These differences are reflected in the fact that while sputum PCR screening is sometimes cost-saving (CS) relative to MMR screening with sputum PCR detection of MDR-TB, it almost always produces a higher health benefit, leading to high confidence that it has a favorable ICER.

b In the table, the term “incremental” refers to comparison between non-dominated strategies and their next best alternative. Sputum PCR screening's costs, QALYs, and ICER are incremental to those of MMR screening with sputum PCR detection of MDR-TB. Dominated strategies cost more and provide less health benefit than an alternative strategy or provide fewer health benefits at a higher cost per health benefit.

c MMR screening with sputum PCR detection of MDR-TB costs less and is more effective than MMR screening, the current status quo in prisons in the FSU. Hence, it dominates the current status quo and is the “reference” strategy for the analysis.

d Combined MMR and symptom screening is dominated via extended dominance—i.e., its ratio of additional costs (US$60,341) to additional QALYs (85) compared to MMR screening with sputum PCR detection of MDR-TB is less favorable than for sputum PCR screening (US$765/QALY versus US$543/QALY). Therefore, if the decision maker is prepared to pay this less favorable, higher amount for additional QALYs from combined MMR and symptom screening, he or she should be prepared to implement sputum PCR screening, since it provides better value for money.

The case finding strategy currently used in most prisons of the FSU—annual MMR screening—was more effective at reducing overall TB and MDR-TB prevalence than strategies based on symptom screening or self-referral only (Table 4; Figure 3). Changing from MMR to symptom screening increased overall TB prevalence at 10 y from 2.74% to 3.39%, with little change in MDR-TB prevalence (0.74% to 0.78%). Changing from MMR to self-referral only (i.e., discontinuing active screening) increased overall TB prevalence at 10 y to 4.28% and MDR-TB prevalence from 0.74% to 0.99%.

The addition of sputum PCR to any screening strategy as a test only for multidrug resistance resulted in reductions in MDR-TB prevalence, though, as expected, used in this way, its effect on overall TB prevalence was small. The preferred use of sputum PCR ultimately depends on an assessment of cost-effectiveness, described below.

### Costs and Cost-Effectiveness

**Figure 4:**
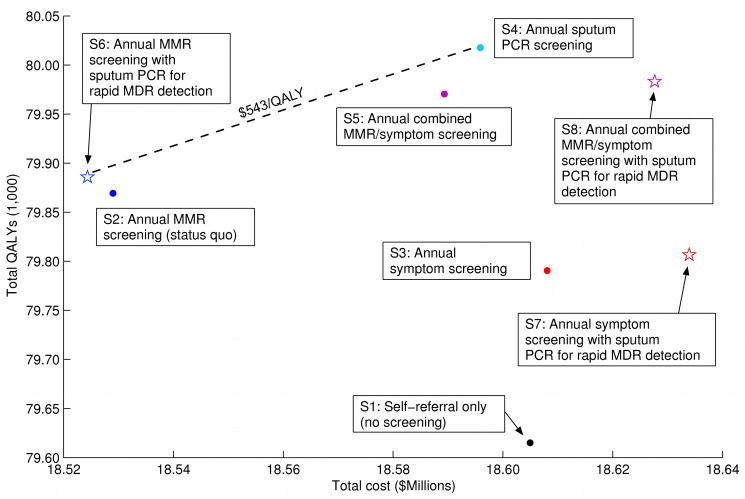
Base case cost-effectiveness frontier. Total costs and total QALYs are shown for each strategy. The cost-effectiveness frontier, illustrated by the dashed line, indicates the strategies with the lowest cost per QALY. The ICER gives the cost in US dollars for each additional QALY gained, as one chooses more costly and effective alternatives along the cost-effectiveness frontier. The black dot denotes no screening; dark blue symbols denote strategies using MMR screening alone; red symbols denote strategies using annual symptom screening alone; light blue symbols denote strategies using sputum PCR screening; purple symbols denote strategies using combined MMR and symptom screening. Star-shaped symbols denote strategies where sputum PCR is used only for rapid MDR-TB detection among individuals who screen positive for TB.

MMR screening with the addition of sputum PCR for rapid MDR-TB detection was the least costly strategy, providing a total cost-savings of US$4,643 over 10 y for our model prison of 1,000 inmates, compared with the currently used strategy of MMR screening alone. The use of sputum PCR as a primary screening tool for TB case detection cost an additional US$71,551 per 1,000 inmates compared to MMR screening with sputum PCR. Using sputum PCR as an annual screening test for all inmates cost US$543 per additional QALY gained compared to MMR screening with sputum PCR (Table 4; Figure 4). Other strategies involving MMR and/or symptom screening, either alone or in combination with sputum PCR, were either more expensive and less effective or less cost-effective than strategies involving sputum PCR alone or MMR screening with sputum PCR (Table 4; Figure 4).

### Country-Specific Analyses

We considered the cost-effectiveness of TB screening and diagnostic strategies separately for prisons in Tajikistan, the Russian Federation, and Latvia, which are characterized by different TB prevalences, MDR-TB prevalences, and per-capita GDPs (Table S2) [1],[13],[19]. Tajikistan and Latvia are small republics spanning the former Soviet region's range of TB prevalences (322 per 100,000 and 55 per 100,000, respectively) and per-capita GDPs (US$1,900 and US$14,600, respectively). The Russian Federation, the most populous former Soviet country (population of 140 million), has a high per-capita GDP (US$15,300) and a TB prevalence of 115 per 100,000.

**Figure 5:**
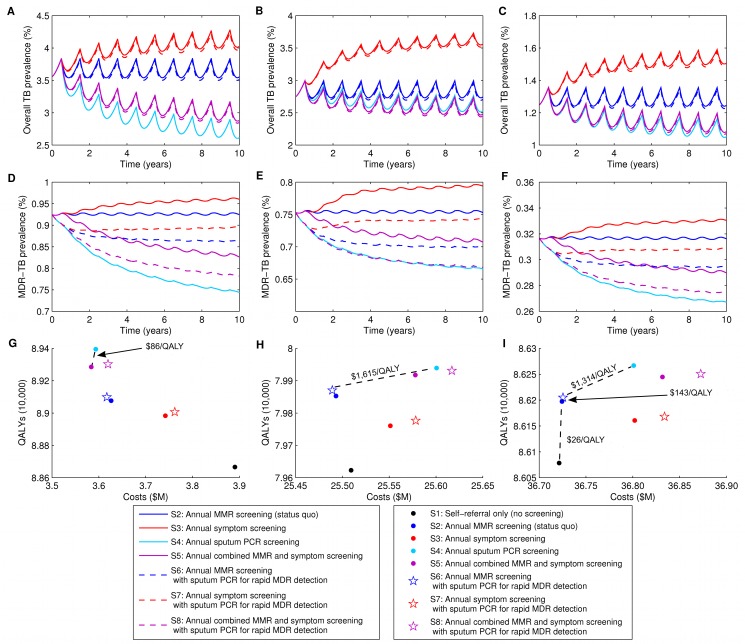
Outcomes for country-specific analysis. Overall TB prevalence rates (A–C), MDR-TB prevalence rates (D–F), and cost-effectiveness frontiers (G–I) over 10 y within prisons in three countries. These outcomes reflect model prisons in Tajikistan (A, D, and G), the Russian Federation (B, E, and H), and Latvia (C, F, and I). Strategy 1 (S1), self-referral only (no screening), is not shown in tracings of overall and MDR-TB prevalence (A–F).

The effectiveness of the evaluated strategies in reducing TB and MDR-TB prevalence in the three FSU countries did not differ substantially from that found in our analysis of the FSU as a whole, despite differing epidemiological situations in these three countries (Table 5; Figure 5). While the relative cost-effectiveness of case detection strategies displayed several notable differences, the main policy finding from our analysis—the cost-effectiveness of sputum PCR as a primary screening tool for case detection—did not change. Specifically, the incremental cost per QALY of the most effective strategy—sputum PCR screening—remained below per-capita GDP in all three countries. The ICERs of more effective strategies were in general higher for the Russian Federation and Latvia than for Tajikistan or the FSU as a whole. Price differences alone do not explain this effect. Of note, the proportion of smear-positive cases was lower in studies of Russian prisons than in studies of prisons elsewhere in the FSU, and in Latvia the prevalence of TB in both the general population and in prisons is relatively low, which may account for the increased cost of strategies that more effectively reduced transmission in these two countries.

**Table 5:** Outcomes for a prison of 1,000 individuals in Tajikistan, the Russian Federation, and Latvia.

Strategy	Total Costs	Total QALYs	Prevalence of TB (Percent)	Prevalence of MDR-TB (Percent)	Strategy on Efficient Frontier	Incremental^a^ Costs	Incremental^a^ QALYs	ICER^a^ (Cost/QALY)
**Tajikistan**								
Combined MMR and symptom screening	$3,583,890	89,285	2.88	0.83	Reference^b^			
Sputum PCR screening	$3,593,364	89,394	2.62	0.74	Non-dominated	$9,474	109	$87
MMR screening with sputum PCR detection of MDR-TB	$3,617,040	89,098	3.51	0.86	Dominated			
Combined MMR and symptom screening with sputum PCR detection of MDR-TB	$3,619,653	89,302	2.85	0.78	Dominated			
MMR screening (status quo)	$3,626,390	89,076	3.55	0.92	Dominated			
Symptom screening	$3,741,989	88,984	4.03	0.96	Dominated			
Symptom screening with sputum PCR detection of MDR-TB	$3,762,149	89,006	3.98	0.90	Dominated			
Self-referral (no screening)	$3,890,535	88,666	5.27	1.27	Dominated			
**Russia**								
MMR screening with sputum PCR detection of MDR-TB	$25,489,211	79,869	2.71	0.70	Reference^b^			
MMR screening (status quo)	$25,493,199	79,852	2.74	0.75	Dominated			
Self-referral (no screening)	$25,509,322	79,624	4.34	0.99	Dominated			
Symptom screening	$25,551,301	79,760	3.56	0.79	Dominated			
Combined MMR and symptom screening	$25,577,974	79,918	2.48	0.71	Extended Dominance^c^			
Symptom screening with sputum PCR detection of MDR-TB	$25,578,348	79,777	3.52	0.74	Dominated			
Sputum PCR screening	$25,600,606	79,940	2.52	0.67	Non-dominated	$111,395	71	$1,569
Combined MMR and symptom screening with sputum PCR detection of MDR-TB	$25,617,006	79,930	2.45	0.67	Dominated			
**Latvia**								
Self-referral (no screening)	$36,721,365	86,079	1.87	0.43	Reference^b^			
MMR screening (status quo)	$36,724,427	86,198	1.25	0.32	Non-dominated	$3,062	119	$26
MMR screening with sputum PCR detection of MDR-TB	$36,725,508	86,206	1.23	0.29	Non-dominated	$1,081	9	$135
Sputum PCR screening	$36,800,972	86,266	1.05	0.27	Non-dominated	$75,464	60	$1,258
Symptom screening	$36,802,038	86,159	1.51	0.33	Dominated			
Combined MMR and symptom screening	$36,831,655	86,244	1.08	0.29	Dominated			
Symptom screening with sputum PCR detection of MDR-TB	$36,833,838	86,167	1.49	0.31	Dominated			
Combined MMR and symptom screening with sputum PCR detection of MDR-TB	$36,872,584	86,249	1.07	0.27	Dominated			

All costs are given in 2009 US dollars. Quality of life weights used for these analyses are shown in Table S8. Shown are TB and MDR-TB prevalence rates, total costs, and total QALYs saved at the end of 10 y as well as ICERs for all eight strategies in three specific countries. Initial TB prevalence rates for Tajikistan, Russian Federation, and Latvia were 3.55%, 2.74%, and 1.25%, respectively. Initial MDR-TB prevalence rates were 0.92%, 0.75%, and 0.32%, respectively.

a In the table, the term “incremental” refers to comparison between non-dominated strategies and their next best alternative. In the cases of Tajikistan and Russia, sputum PCR screening is compared to combined MMR and symptom screening and MMR screening with sputum PCR detection of MDR-TB, respectively. In the case of Latvia, multiple strategies are non-dominated and hence are each compared to their next best alternative (i.e., the immediately preceding strategy in the table). Dominated strategies cost more and provide less health benefit than an alternative strategy.

b In each country, the marked strategy costs less and is more effective than MMR screening, the current status quo in prisons in the FSU. Hence, it dominates the current status quo and is then the reference strategy for the analysis.

c Combined MMR and symptom screening is dominated via extended dominance—i.e., its ratio of additional costs (US$88,763) to additional QALYs (49) compared to MMR screening with sputum PCR detection of MDR-TB is less favorable than for sputum PCR screening (US$1,811/QALY versus US$1,569/QALY). Therefore, if the decision maker is prepared to pay this less favorable, higher amount for additional QALYs from combined MMR and symptom screening, he or she should be prepared to implement sputum PCR screening, since it provides better value for money.

### Sensitivity and Uncertainty Analyses

We performed a series of sensitivity analyses for our model, in order to evaluate the impact of uncertainty on our findings. Sputum PCR used as a primary screening tool remained cost-effective for virtually all parameter combinations evaluated.

In all one-way sensitivity analyses, the cost per QALY gained for using sputum PCR as an annual primary screening tool for TB among the general prison population remained below US$10,561 per QALY, the average per-capita GDP of former Soviet countries (Table S3). The cost per QALY gained for sputum PCR was lower and more favorable when the rate of reinfection among those latently infected or recovered was higher (for *ν* = 0.20, the ICER was US$1,798/QALY; for *ν* = 0.60, the ICER was US$263/QALY), when the cost of performing sputum PCR was lower (for a per-test cost of US$18.06, the ICER was US$166/QALY; for a per-test cost of US$30.09, the ICER was US$1,241/QALY), when the proportion of individuals who rapidly develop active disease was higher (for *q* = 0.13, the ICER was US$1,076/QALY; for *q* = 0.21, the ICER was US$244/QALY), and when the contact rate for non-MDR-TB was higher than that of MDR-TB (for β_d_ = 5.25, the ICER was US$995/QALY; for β_d_ = 8.75, the ICER was US$225/QALY) (Table S3). For some parameter combinations, MMR alone without the use of sputum PCR for MDR-TB detection was the least costly strategy. Combined MMR and symptom screening was a cost-effective strategy in two scenarios: when there was a high cost of sputum PCR (US$30.09) and when there was a very high specificity for the process of MDR-TB detection under current clinical circumstances (100%). Self-referral alone was cost-effective with only one parameter combination: when the relative rate of reinfection was 0.20. No other strategy appeared cost-effective in univariate analyses.

**Figure 6:**
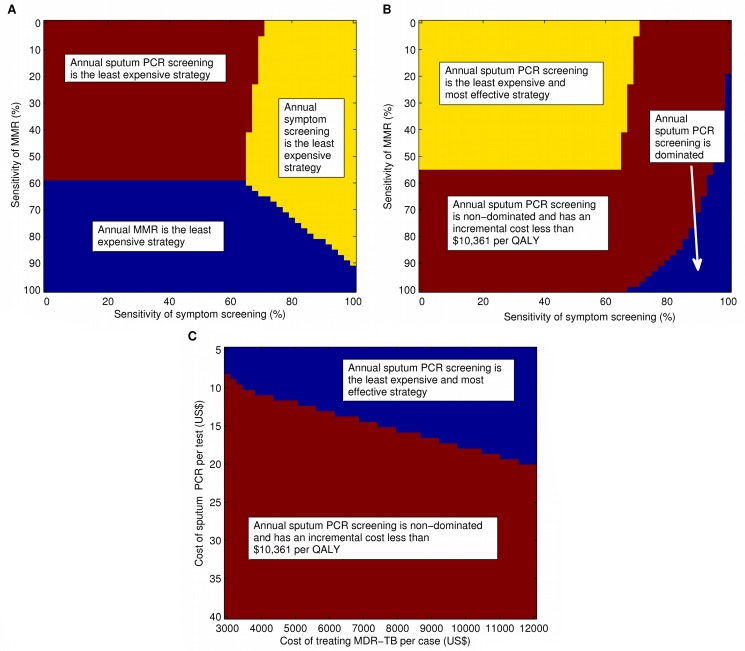
Results of two-way sensitivity analyses. (A) Test sensitivities of MMR and of symptom screening are varied from 0% to 100%. Colored regions indicate combinations of test sensitivities for which sputum PCR screening (maroon), symptom screening (yellow), and MMR (blue) are the least costly of the three screening strategies evaluated. (B) Test sensitivities of MMR and of symptom screening are varied from 0% to 100%. Colored regions indicate the ICER of sputum PCR screening compared with the next best strategy, divided into the following: cost-saving (yellow), non-dominated and ICER<US$10,561 (maroon), and dominated (blue); the ICER did not exceed US$10,561 over the ranges evaluated. (C) The per-test cost of sputum PCR and the per-case cost of treatment of MDR-TB are varied from US$5 to US$40 and from US$3,000 to US$12,000 respectively. Colored regions indicate the ICER of sputum PCR screening compared with the next best strategy, divided into the following: cost-saving (blue) and non-dominated and ICER<US$10,561 (maroon); the ICER did not exceed US$10,561 over the ranges evaluated.

We performed two-way sensitivity analyses to evaluate the cost-effectiveness of our three main screening tools over the potential range of test sensitivities for MMR and symptom screening, as the characteristics of these tests may vary widely with local conditions (Figure 6). In these analyses, sputum PCR becomes cost-saving if the sensitivity of MMR falls below approximately 60%, and with symptom screening having a sensitivity of less than 65%. Furthermore, these analyses show that only when the sensitivity of both MMR and symptom screening are very high (both >80%) is sputum PCR dominated by strategies based on these methods. When sputum PCR screening is excluded from the analysis, a similar two-way sensitivity analysis shows that the ICER of combining MMR and symptom screening into one annual case finding activity remains below the average per-capita GDP of former Soviet countries (US$10,561) for nearly the entire potential range of sensitivities.

In an additional two-way sensitivity analysis, we evaluated the cost-effectiveness of sputum PCR screening for a wide range of costs for both MDR-TB treatment and for sputum PCR itself (Figure 6). In this analysis, the incremental cost per QALY of sputum PCR screening remained below US$10,561, even at a cost of US$40 per test. When the cost of treating MDR-TB is high (US$12,000), sputum PCR screening becomes cost-saving at a price of US$20 per test. If the cost of treating MDR-TB is low (US$3,000), sputum PCR screening is only cost-saving when its cost is less than US$8.

We also evaluated the differential cost-effectiveness of the case detection strategies under consideration in a number of alternative plausible scenarios (Table S4). In our base case, we assumed that the contact rates for both MDR-TB and non-MDR-TB were similar to those found in studies of non-incarcerated populations. In a situational analysis in which the contact rates for both forms of TB were twice as high as previously estimated for civilian populations (14 contacts per infectious case per year leading to new infection), sputum PCR screening was both more effective and less expensive than all other strategies under consideration (Table S4). Similarly, narrowing screening intervals from yearly to every 6 mo produced substantial reductions in TB and MDR-TB prevalence for any given case finding strategy, but increased the incremental cost of using more sensitive methods such as sputum PCR screening. When performed twice annually, sputum PCR screening cost US$2,602 per additional QALY gained compared to MMR screening with sputum PCR for MDR-TB detection (Table S4). In a situational analysis modeling a 6-wk drug shortage after 5 y, the relative cost-effectiveness of alternative strategies at the end of 10 y was minimally affected (Table S4). When MMR was modeled to have the same sensitivity for smear-negative TB cases as for smear-positive TB cases, the magnitude of the health benefits from each strategy was similar to the base case, as was each strategy's relative ranking, with sputum PCR screening costing US$490 per additional QALY gained compared to MMR screening with sputum PCR detection of MDR-TB (Table S4). When we considered an alternative assumption that reinfection with non-MDR-TB of individuals with prior MDR-TB infection was possible, in addition to reinfection with MDR-TB as before (Figure S4), and also assumed that those individuals reinfected with non-MDR-TB would be treated with first-line therapy, we found that sputum PCR screening cost US$552 per additional QALY gained relative to MMR screening with sputum PCR detection of MDR-TB (Table S4).

**Figure 7:**
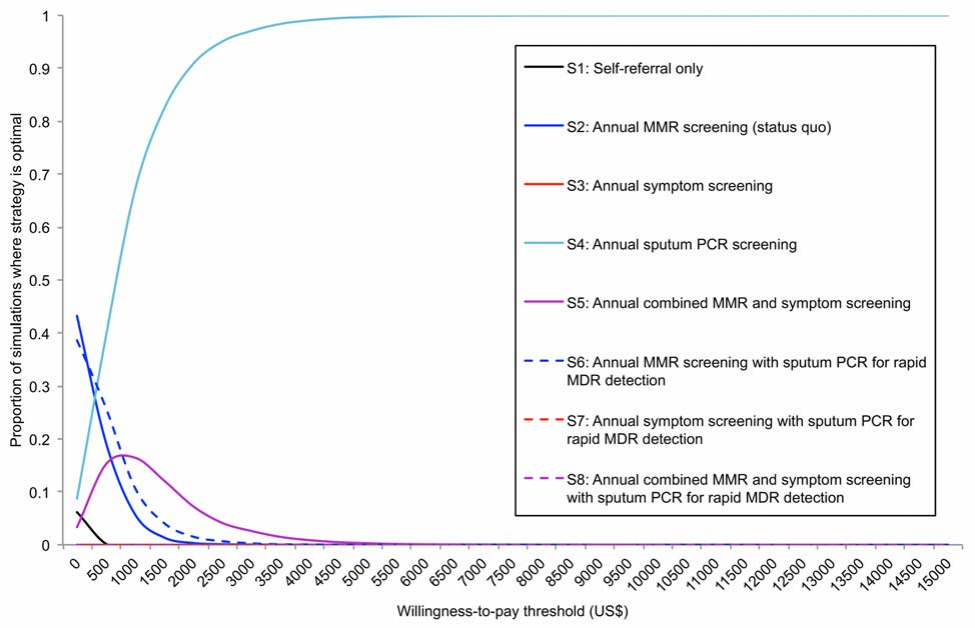
Results of the probabilistic sensitivity analysis. Ten thousand parameter combinations were randomly selected, and the NMB was calculated for each strategy in each parameter combination. The likelihood that each strategy is preferred (has the highest NMB) at willingness-to-pay thresholds from US$0 to US$15,000 is shown.

To reflect the overall uncertainty in all model inputs, we conducted a probabilistic sensitivity analysis (Figure 7). Above a willingness-to-pay threshold of US$2,500 per QALY (approximately 25% of per-capita GDP in the FSU), sputum PCR used as a primary screening tool was considered cost-effective in more than 95% of the 10,000 parameter combinations sampled.

## Discussion

The use of sputum PCR as an annual, primary screen for TB in the general prison population of former Soviet countries was the most effective strategy for reducing both TB and MDR-TB prevalence, and provided health benefit at a cost well below their average per-capita GDP. The Commission on Macroeconomics and Health considers an intervention whose cost per QALY gained is below a country's per-capita GDP to be very cost-effective [30]. Though it would require significant investment, expanding case finding efforts in prisons with high prevalence of MDR-TB to include screening with sputum PCR will likely lead to substantially improved TB disease control, with the increased costs offset by decreased expenditures on MDR-TB treatment.

In settings where the implementation of sputum PCR screening is not feasible, combined MMR and symptom screening is a cost-effective alternative that produces substantial reductions in TB and MDR-TB prevalence, at low cost per QALY compared with per-capita GDP. In Tajikistan, where the cost of labor is low, this strategy was cost-saving over the 10-y time horizon considered. In other settings, MMR-based strategies were cost-saving.

The highly attractive cost-per-QALY-gained profile of sputum PCR is driven by three features of the populations and settings we consider: the high prevalence of TB, the high proportion of MDR-TB cases, and the availability of both first-line and second-line treatment regimens. In our country-specific analysis for Tajikistan, the poorest of the three countries specifically modeled, the incremental cost per QALY of sputum PCR screening was lower than for Russia, Latvia, or the FSU in general, despite the higher estimated per-test cost there. This is explained by the higher than average prevalence of TB and MDR-TB in Tajikistan, relative to the other settings. In prison settings with TB/MDR-TB prevalence lower than those considered here, the incremental cost per QALY gained for sputum PCR screening is likely substantially higher. Importantly, even in lower prevalence settings, if the cost of MDR-TB therapy could be reduced, the ICER of sputum PCR screening would be further improved: reducing the costs of MDR-TB drugs themselves and avoiding overuse of precautionary hospitalization of MDR-TB cases are potentially policy-relevant approaches to achieving lower MDR-TB therapy costs. Because much of the benefits of sputum PCR screening come from detecting MDR-TB cases that can be effectively treated, it is not appropriate to apply our findings to prison settings without a functioning MDR-TB treatment program.

Studying disease control in places of incarceration presents important challenges. Security concerns often predominate over public health threats in the daily operations of prisons and other detention facilities, and the public perception of inmates can lower prison health as a research priority for government funding agencies. Therefore, a major limitation of our study was the availability of primary data regarding TB epidemiology and control in prisons in the FSU. This required us to make multiple simplifying assumptions about the biology and treatment of TB and to draw from a variety of heterogeneous data sources to estimate model inputs. Despite this heterogeneity and uncertainty, our many sensitivity and scenario analyses suggest that the main results—that sputum PCR is cost-effective when used as a primary screening tool for TB in prisons of high prevalence—was robust. However, time-series data against which to calibrate or validate the model were not available, so we cannot be sure which parameter combinations best match reality. While we performed multiple sensitivity and uncertainty analyses, including probabilistic sensitivity analyses, for which the large majority supported the robustness of the findings in the main analysis, the potential still remains that the type of correlation structure present in the joint posterior distribution of model parameters determined via empirical calibration could lead to different conclusions regarding robustness.

Very little prospective data exist regarding the test characteristics of MMR. We conducted a systematic review of the literature on MMR to identify all relevant articles for our estimates of its sensitivity and specificity (Text S1). We also conducted two-way sensitivity analyses across the entire range of possible values for MMR's sensitivity to determine thresholds for cost-effectiveness. In these analyses, MMR screening maintains a favorable ratio cost per QALY gained as long as it maintains a sensitivity of >60% for pulmonary TB (Figure 6). Finally, because of the possibility that MMR's performance differs between smear-positive and smear-negative cases in ways that are difficult to adjust for via verification bias adjustments, we conducted two sensitivity analyses where we assumed either that (1) MMR sensitivity for smear-negative cases was equal to that of smear-positive cases or (2) MMR sensitivity for smear-positive cases was at the upper bound of its confidence interval and smear-negative sensitivity was at the lower bound of its confidence interval. In both cases, the results remained consistent with those in the main analysis.

Our estimates of test characteristics are further complicated by the lack of a functional “gold standard” for TB diagnosis. While culture positivity is considered to be the “gold standard” case definition for epidemiological studies, a significant proportion of smear-negative TB cases as defined clinically on the basis of nonresponse to broad spectrum antibiotics are culture-negative. More invasive methods such as bronchoalveolar lavage can show a meaningful proportion of these individuals to have bacteriologically positive disease [31]–[34]. Since a clinical diagnosis is usually used as the basis for treatment, we included a proportion of such “abacillary cases” in our estimates of sensitivity and specificity for the diagnostic tools considered in the analysis (Text S1). A scenario analysis in which test characteristic estimates included only bacteriologically positive cases resulted in the same conclusions as our main analysis (Table S4).

Our study did not explicitly model HIV, given data limitations and the complexity of HIV-TB co-infections. However, it is well known that HIV affects the susceptibility for and clinical presentation of TB in ways relevant to the screening methods we have examined. In most former Soviet republics, estimates of HIV prevalence in places of incarceration range from 0% to 4.76%, but higher rates have been reported in select prisons in Ukraine and the Baltic states [35]. Our analysis is likely less accurate for these settings, though it is difficult to predict how including HIV might have affected our results, given that HIV disease can impact both the radiographic appearance and the bacillary load of sputum in individuals co-infected with TB [36].

Another important limitation of our model is the absence of a separate compartment for individuals who default from treatment. In prisons of the FSU, treatment default is most often due either to early release or to transfer to another facility. In our model, default was incorporated into treatment outcomes by assuming these individuals would remain on treatment until treatment success, treatment failure, death, or release. A scenario analysis in which treatment outcomes immediately after release were worse than those reported—accounting for a lapse in treatment—did not substantially impact the results (Table S4).

Our estimates of the cost-effectiveness of more sensitive screening strategies are likely conservative because, while we did model morbidity, mortality, and costs resulting from active disease occurring after release from prison, we did not model post-release transmission in the general, non-prison population and do not capture the consequent benefits and averted costs of better TB control in prisons reducing such transmission. Therefore, the cost-effectiveness of strategies reducing TB and MDR-TB prevalence among inmates may be underestimated.

As global TB control efforts expand to cover comprehensive treatment for MDR-TB, the efficient use of scarce healthcare resources is paramount. The use of interventions, including sputum PCR for case finding and rapid MDR-TB detection, that maximally interrupt the cycle of transmission in prisons where TB is prevalent and MDR-TB strains are concentrated may save resources while promoting a culture of human rights for prison residents and averting preventable deaths both inside and outside prison walls.

## Supporting Information

Figure S1 **Decision model for cost-effectiveness analysis.**
(TIF)

Figure S2 **Transmission model for natural history, diagnosis, and treatment of TB.**
(TIF)

Figure S3 **Simplified Markov models for remaining discounted life expectancy, quality-adjusted life expectancy, and TB-related costs after release from prison.**
(TIF)

Figure S4 **Scenario analysis in which reinfection with non-MDR-TB may occur among those previously treated for MDR-TB.**
(TIF)

Table S1 **Definitions and values of model parameters.**
(DOC)

Table S2 **Demographic and epidemiologic characteristics of Tajikistan, Russian Federation, and Latvia.**
(DOC)

Table S3 **Results of univariate sensitivity analysis.**
(DOC)

Table S4 **Outcomes for selected alternative scenarios.**
(DOC)

Table S5 **Health states and their transitions.**
(DOC)

Table S6 **Data used to estimate test characteristics of MMR and symptom screening.**
(DOC)

Table S7 **Cost components.**
(DOC)

Table S8 **Health state utility weights.**
(DOC)

Text S1 **Technical appendix.**
(DOC)

## Abbreviations

DOTS: Directly Observed Therapy Short-Course
FSU: former Soviet Union
GDP: gross domestic product
ICER: incremental cost-effectiveness ratio
MDR-TB: multidrug-resistant tuberculosis
MMR: mass miniature radiography
NMB: net monetary benefit
QALY: quality-adjusted life year
TB: tuberculosis
WHO: World Health Organization

## References

[1] World Health Organization (2010) Global tuberculosis control 2010. Geneva: World Health Organization.

[2] Coninx R, Maher D, Reyes H, Grzemska M (2000) Tuberculosis in prisons in countries with high prevalence. BMJ 320: 440–442. 10669453 10.1136/bmj.320.7232.440 PMC1117551

[3] Stuckler D, Basu S, McKee M, King L (2008) Mass incarceration can explain population increases in TB and multidrug-resistant TB in European and central Asian countries. Proc Natl Acad Sci U S A 105: 13280–13285 doi:10.1073/pnas.0801200105. 18728189 10.1073/pnas.0801200105 PMC2533181

[4] Dara M, Grzemska M, Kimerling ME, Reyes H, Zagorskiy A (2009) Guidelines for control of tuberculosis in prisons. Washington (District of Columbia): U.S. Agency for International Development.

[5] Bone A, Aerts A, Grzemska M, Kimerling M, Kluge H, et al.. (2000) Tuberculosis control in prisons: a manual for programme managers. Geneva: World Health Organization.

[6] Belov IB, Kitaev VM (2000) [Comparative assessment of diagnostic capacities of thoracic fluorography, film x-ray study and small-dose digital x-ray study.]. Probl Tuberk 6: 23–27. 11209739

[7] Legrand J, Sanchez A, Le Pont F, Camacho L, Larouze B (2008) Modeling the impact of tuberculosis control strategies in highly endemic overcrowded prisons. PLoS ONE 3: e2100 doi:10.1371/journal.pone.0002100. 18461123 10.1371/journal.pone.0002100 PMC2324198

[8] van der Werf MJ, Enarson DA, Borgdorff MW (2008) How to identify tuberculosis cases in a prevalence survey. Int J Tuberc Lung Dis 12: 1255–1260. 18926034

[9] Shin SS, Pasechnikov AD, Gelmanova IY, Peremitin GG, Strelis AK, et al (2006) Treatment outcomes in an integrated civilian and prison MDR-TB treatment program in Russia. Int J Tuberc Lung Dis 10: 402–408. 16602404

[10] Gold MR, Siegel JE, Russell LB, Weinstein MC, editors (1996) Cost-effectiveness in health and medicine. New York: Oxford University Press.

[11] Sohn H, Minion J, Albert H, Dheda K, Pai M (2009) TB diagnostic tests: how do we figure out their costs? Expert Rev Anti Infect Ther 7: 723–733 doi:10.1586/eri.09.52. 19681700 10.1586/eri.09.52

[12] International Labour Organization (2011) LABORSTA Internet: statistics by country [database]. Geneva: International Labour Organization.

[13] World Health Organization (2011) Unit costs for patient services. CHOosing Interventions that are Cost Effective (WHO-CHOICE). Geneva: World Health Organzation.

[14] Vassall A, van Kampen S, Sohn H, Michael JS, John KR, et al (2011) Rapid diagnosis of tuberculosis with the Xpert MTB/RIF assay in high burden countries: a cost-effectiveness analysis. PLoS Med 8: e1001120 doi:10.1371/journal.pmed.1001120. 22087078 10.1371/journal.pmed.1001120 PMC3210757

[15] Sackett DL, Torrance GW (1978) The utility of different health states as perceived by the general public. J Chronic Dis 31: 697–704. 730825 10.1016/0021-9681(78)90072-3

[16] Guo N, Marra CA, Marra F, Moadebi S, Elwood RK, et al (2008) Health state utilities in latent and active tuberculosis. Value Health 11: 1154–1161 doi:10.1111/j.1524-4733.2008.00355.x. 18489493 10.1111/j.1524-4733.2008.00355.x

[17] Bobrik A, Danishevski K, Eroshina K, McKee M (2005) Prison health in Russia: the larger picture. J Public Health Policy 26: 30–59 doi:10.1057/palgrave.jphp.3200002. 15906874 10.1057/palgrave.jphp.3200002

[18] World Health Organization (2011) Global Health Observatory Data Repository: mortality and burden of disease—life expectancy: life tables [database]. Available: http://apps.who.int/gho/data/. Accessed 21 June 2011.

[19] World Health Organization (2011) Global Health Observatory Data Repository: health systems—health financing [database]. Available: http://apps.who.int/gho/data/. Accessed 21 June 2011.

[20] Winqvist N, Björk J, Miörner H, Björkman P (2011) Long-term course of Mycobacterium tuberculosis infection in Swedish birth cohorts during the twentieth century. Int J Tuberc Lung Dis 15: 736–740 doi:10.5588/ijtld.10.0683. 21575291 10.5588/ijtld.10.0683

[21] Aerts A, Habouzit M, Mschiladze L, Malakmadze N, Sadradze N, et al (2000) Pulmonary tuberculosis in prisons of the ex-USSR state Georgia: results of a nation-wide prevalence survey among sentenced inmates. Int J Tuberc Lung Dis 4: 1104–1110. 11144451

[22] Balabanova Y, Drobniewski F, Fedorin I, Zakharova S, Nikolayevskyy V, et al (2006) The Directly Observed Therapy Short-Course (DOTS) strategy in Samara Oblast, Russian Federation. Respir Res 7: 44 doi:10.1186/1465-9921-7-44. 16556324 10.1186/1465-9921-7-44 PMC1440858

[23] Bonnet M, Sizaire V, Kebede Y, Janin A, Doshetov D, et al (2005) Does one size fit all? Drug resistance and standard treatments: results of six tuberculosis programmes in former Soviet countries. Int J Tuberc Lung Dis 9: 1147–1154. 16229227

[24] Drobniewski F, Tayler E, Ignatenko N, Paul J, Connolly M, et al (1996) Tuberculosis in Siberia: 1. an epidemiological and microbiological assessment. Tuber Lung Dis 77: 199–206. 8758101 10.1016/s0962-8479(96)90001-5

[25] Krivonos PS, Avdeev GS (2005) [Tuberculosis control in the penitentiaries of the Republic of Belarus: state-of-the-art and prospects.]. Probl Tuberk Bolezn Legk 5: 22–25. 15988973

[26] Nechaeva OB, Skachkova EI, Podymova AS (2005) [Tuberculosis in the prisons of the Sverdlovsk Region.]. Probl Tuberk Bolezn Legk 5: 16–18. 15988971

[27] Republican Center for Tuberculosis Control (2009) [Statistical information on tuberculosis for years 2007–2008 in the Republic of Tajikistan.] Dushanbe (Tajikistan): Ministry of Health, Republic of Tajikistan.

[28] Coker RJ, Dimitrova B, Drobniewski F, Samyshkin Y, Balabanova Y, et al (2003) Tuberculosis control in Samara Oblast, Russia: institutional and regulatory environment. Int J Tuberc Lung Dis 7: 920–932. 14552561

[29] Leimane V, Leimans J (2006) Tuberculosis control in Latvia: integrated DOTS and DOTS-plus programmes. Euro Surveill 11: 29–33. 16567876

[30] World Health Organization (2011) CHOosing Interventions that are Cost Effective (WHO-CHOICE): cost-effectiveness thresholds. Available: http://www.who.int/choice/costs/CER_thresholds/en/index.html. Accessed 21 June 2011.

[31] Purohit S, Sisodia R, Gupta P, Sarkar S, Sharma T (1983) Fiberoptic bronchoscopy in diagnosis of smear negative pulmonary tuberculosis. Lung India 1: 143–146.

[32] de Gracia J, Curull V, Vidal R, Riba A, Orriols R, et al (1988) Diagnostic value of bronchoalveolar lavage in suspected pulmonary tuberculosis. Chest 93: 329–332. 3123151 10.1378/chest.93.2.329

[33] Wongthim S, Udompanich V, Limthongkul S, Charoenlap P, Nuchprayoon C (1989) Fiberoptic bronchoscopy in diagnosis of patients with suspected active pulmonary tuberculosis. J Med Assoc Thai 72: 154–159. 2738497

[34] Saglam L, Akgun M, Aktas E (2005) Usefulness of induced sputum and fibreoptic bronchoscopy specimens in the diagnosis of pulmonary tuberculosis. J Int Med Res 33: 260–265. 15790139 10.1177/147323000503300215

[35] Dolan K, Kite B, Black E, Aceijas C, Stimson GV (2007) HIV in prison in low-income and middle-income countries. Lancet Infect Dis 7: 32–41 doi:10.1016/S1473-3099(06)70685-5. 17182342 10.1016/S1473-3099(06)70685-5

[36] World Health Organization Stop TB Department, World Health Organization Department of HIV/AIDS, World Health Organization Department of Child and Adolescent Health and Development (2004) TB/HIV: a clinical manual, 2nd edition. Geneva: World Health Organization.

[37] Datta M, Radhamani MP, Sadacharam K, Selvaraj R, Rao DL, et al (2001) Survey for tuberculosis in a tribal population in North Arcot District. Int J Tuberc Lung Dis 5: 240–249. 11326823

[38] Gopi PG, Subramani R, Radhakrishna S, Kolappan C, Sadacharam K, et al (2003) A baseline survey of the prevalence of tuberculosis in a community in south India at the commencement of a DOTS programme. Int J Tuberc Lung Dis 7: 1154–1162. 14677890

[39] Lewis JJ, Charalambous S, Day JH, Fielding KL, Grant AD, et al (2009) HIV infection does not affect active case finding of tuberculosis in South African gold miners. Am J Respir Crit Care Med 180: 1271–1278 doi:10.1164/rccm.200806-846OC. 19745207 10.1164/rccm.200806-846OC PMC2796737

[40] Churchyard GJ, Fielding KL, Lewis JJ, Chihota VN, Hanifa Y, et al (2010) Symptom and chest radiographic screening for infectious tuberculosis prior to starting isoniazid preventive therapy: yield and proportion missed at screening. AIDS 24 (Suppl 5) S19–S27 doi:10.1097/01.aids.0000391018.72542.46. 21079424 10.1097/01.aids.0000391018.72542.46

[41] den Boon S, White NW, van Lill SWP, Borgdorff MW, Verver S, et al (2006) An evaluation of symptom and chest radiographic screening in tuberculosis prevalence surveys. Int J Tuberc Lung Dis 10: 876–882. 16898372

[42] Boehme CC, Nabeta P, Hillemann D, Nicol MP, Shenai S, et al (2010) Rapid molecular detection of tuberculosis and rifampin resistance. N Engl J Med 363: 1005–1015 doi:10.1056/NEJMoa0907847. 20825313 10.1056/NEJMoa0907847 PMC2947799

[43] Matthys F, Rigouts L, Sizaire V, Vezhnina N, Lecoq M, et al (2009) Outcomes after chemotherapy with WHO category II regimen in a population with high prevalence of drug resistant tuberculosis. PLoS ONE 4: e7954 doi:10.1371/journal.pone.0007954. 19956770 10.1371/journal.pone.0007954 PMC2776350

[44] Bonnet M, Pardini M, Meacci F, Orrù G, Yesilkaya H, et al (2011) Treatment of tuberculosis in a region with high drug resistance: outcomes, drug resistance amplification and re-infection. PLoS ONE 6: e23081 doi:10.1371/journal.pone.0023081. 21886778 10.1371/journal.pone.0023081 PMC3160294

[45] Lalor MK, Allamuratova S, Tiegay Z, Khamraev AK, Greig J, et al.. (2011) Treatment outcomes in multi drug resistant TB patients in Uzbekistan [abstract]. 42nd Union World Conference on Lung Health; 26–30 October 2011; Lille, France.

