# Supplementary material for: Screening and Rapid Molecular Diagnosis of Tuberculosis in Prisons in Russia and Eastern Europe: A Cost-Effectiveness Analysis
Source: PLoS Med. 2012 Nov 27;9(11):e1001348. doi: 10.1371/journal.pmed.1001348 (PMC3507963; doi:10.1371/journal.pmed.1001348)
Supplement: Table S1 — Definitions and values of model parameters. (DOC) [file pmed.1001348.s005.doc]

| **Table S1.** Definitions and values of model parameters | | | | |
| --- | --- | --- | --- | --- |
| *(Refer to Figure S1)* | | | | |
| Parameter | Definition | Value | Range | References |
| q | Proportion of new infections that progress rapidly to active disease | 0.17 | [0.13-0.21]† | [7,46,47] |
| ν | Relative rate of re-infection among latently-infected and recovered individuals. | 0.48 | [0.25-0.60] | [59,60] |
| γ_1_ | Annual rate of delayed progression from latent to active disease | 0.00065 | [4.9 X 10^-4^-8.2 X 10^-4^]† | [7,46–49] |
| γ_2_ | Annual rate of rapid progression from latent to active disease | 0.32 | [0.24-0.40]† | [7,46–49] |
| ρ/ χ | Proportion of cases that are smear-positive | 0.58 | [0.55-0.60] | [20,21,26,61] |
| φ_n_/ φ_p_ | Annual rate of detection via self-referral | 0.18^a^ | [0.13-0.23]† | [24] |
| μ | Annual rate of conversion from smear-negative to smear-positive | 0.016 | [0.012-0.020]† | [7,47,62] |
| κ | Annual rate of “self-cure” in which TB resolves without treatment (not shown) | 0 .058 | [0.44-0.73]† | [7] |
| π_d_ | Annual treatment success rate for non-MDR TB | 1.79 | [0.74-2.01] | [22,43] |
| τ_d_ | Annual treatment failure rate for non-MDR TB | 0.059 | [0.038-0.081] | [22,43] |
| π_m_ | Annual treatment success rate for MDR TB | 0.51 | [0.48-0.56] | [9,28,44,45] |
| τ_m_ | Annual treatment failure rate for MDR TB | 0.17 | [0.15-0.19] | [9,28,44,45] |
| ζ1 | Annual death rate from non-infectious causes in all health states | 0.0024 | [1.3 X 10^-3^-  3.0 X 10^-3^]† | [16] |
| ζ2_d/m_ | Annual death rate associated with untreated smear-negative TB | 0.11 | [0.079-0.13]† | *Assumed to be ½ of* ζ3_d/m_ |
| ζ3_d/m_ | Annual death rate associated with untreated smear-positive TB | 0.21 | [0.16-0.26]† | [7,46,47] |
| D | Coefficient of relative infectivity of smear negative cases | 0.22 | [0.20-0.28] | [63–65] |
| z_1_ | Annual relapse rate to active non-MDR TB among previously treated for non-MDR TB | 0.13 | [0.10-0.24] | [43,66] |
| z_2_ | Annual relapse rate to MDR-TB among those previously treated for non-MDR TB | 0.053 | [0.026-0.11] | [43,66] |
| z_3_ | Annual relapse rate to active disease among those previously treated for MDR-TB | 0.19 | [0.33-1.32] | [43,66] |
| β_d_/ β_m_ | Contacts per infectious non-MDR TB case per year leading to infection | 7 | [5.25-8.75]† | [19,46,62] |
| ε_n_/ ε_p_ | Annual rate at which MDR cases are started on DOTS-plus therapy in without sputum PCR | 5.15 | [3.86-6.44]† | *Assumed 75% will transition in < 14 weeks* |
| PrL | Prevalence of latent TB infection among the general non-prison population | 0.53* | [0.23-0.63]* | [54] |

^a^ When no screening step was applied, the annual rate of self-referral rises to 0.36 for smear-negative cases and 0.69 for smear-positive disease.

† Estimated subjectively

* Rate varied manually to match the prevalence of active disease within prisons to observed rates in published data (Text S1).

**References**

46. Resch SC, Salomon JA, Murray M, Weinstein MC (2006) Cost-effectiveness of treating multidrug-resistant tuberculosis. PLoS Med 3: e241. doi:10.1371/journal.pmed.0030241.

47. Murray CJ, Salomon JA (1998) Modeling the impact of global tuberculosis control strategies. Proc Natl Acad Sci USA 95: 13881–13886.

48. Cohen T, Colijn C, Finklea B, Murray M (2007) Exogenous re-infection and the dynamics of tuberculosis epidemics: local effects in a network model of transmission. J R Soc Interface 4: 523–531. doi:10.1098/rsif.2006.0193.

49. Colijn C, Cohen T, Murray M (2006) Mathematical models of tuberculosis: accomplishments and future challenges World Scientific.

54. Drobniewski F, Balabanova Y, Zakamova E, Nikolayevskyy V, Fedorin I (2007) Rates of latent tuberculosis in health care staff in Russia. PLoS Med 4: e55. doi:10.1371/journal.pmed.0040055.

59. Sutherland I, Svandová E, Radhakrishna S (1982) The development of clinical tuberculosis following infection with tubercle bacilli. 1. A theoretical model for the development of clinical tuberculosis following infection, linking from data on the risk of tuberculous infection and the incidence of clinical tuberculosis in the Netherlands. Tubercle 63: 255–268.

60. Vynnycky E, Fine PE (1997) The natural history of tuberculosis: the implications of age-dependent risks of disease and the role of reinfection. Epidemiol Infect 119: 183–201.

61. Toungoussova OS, Mariandyshev A, Bjune G, Sandven P, Caugant DA (2003) Molecular epidemiology and drug resistance of Mycobacterium tuberculosis isolates in the Archangel prison in Russia: predominance of the W-Beijing clone family. Clin Infect Dis 37: 665–672. doi:10.1086/377205.

62. Blower SM, McLean AR, Porco TC, Small PM, Hopewell PC, et al. (1995) The intrinsic transmission dynamics of tuberculosis epidemics. Nat Med 1: 815–821.

63. Behr MA, Warren SA, Salamon H, Hopewell PC, Ponce de Leon A, et al. (1999) Transmission of Mycobacterium tuberculosis from patients smear-negative for acid-fast bacilli. Lancet 353: 444–449.

64. Diel R, Loddenkemper R, Meywald-Walter K, Gottschalk R, Nienhaus A (2009) Comparative performance of tuberculin skin test, QuantiFERON-TB-Gold In Tube assay, and T-Spot.TB test in contact investigations for tuberculosis. Chest 135: 1010–1018. doi:10.1378/chest.08-2048.

65. Tostmann A, Kik SV, Kalisvaart NA, Sebek MM, Verver S, et al. (2008) Tuberculosis transmission by patients with smear-negative pulmonary tuberculosis in a large cohort in the Netherlands. Clin Infect Dis 47: 1135–1142. doi:10.1086/591974.

66. Cox H, Kebede Y, Allamuratova S, Ismailov G, Davletmuratova Z, et al. (2006) Tuberculosis recurrence and mortality after successful treatment: impact of drug resistance. PLoS Med 3: e384. doi:10.1371/journal.pmed.0030384.
