# Supplementary material for: Screening and Rapid Molecular Diagnosis of Tuberculosis in Prisons in Russia and Eastern Europe: A Cost-Effectiveness Analysis
Source: PLoS Med. 2012 Nov 27;9(11):e1001348. doi: 10.1371/journal.pmed.1001348 (PMC3507963; doi:10.1371/journal.pmed.1001348)
Supplement: Table S2 — Demographic and epidemiologic characteristics of Tajikistan, Russian Federation, and Latvia. (DOC) [file pmed.1001348.s006.doc]

| **Table S2.** Demographic and epidemiologic characteristics of Tajikistan, Russian Federation and Latvia. | | | |
| --- | --- | --- | --- |
|  | Tajikistan | Russia | Latvia |
| Prevalence of TB (all forms) |  |  |  |
| General population [1] | 322 per 100,000 | 115 per 100,000 | 55 per 100,000 |
| Prison settings [27]; [23,67,68] (AIDS Foundation East-West, unpublished data); [28] | 3.75% | 2.87% | 1.25% |
| Proportion of cases that are MDR |  |  |  |
| General population [1] | 18.3% | 17.7% | 14.5% |
| Prison settings [22,61,69,70]; [28] | No data | 35.5% | 13.6% |
| Proportion of smear-positive cases |  |  |  |
| General population [1] | 51% | 31% | 51% |
| Prison settings [26]; [21,61] | 83% | 35% | No data |
| Baseline health expenditure [18] | $13 | $306 | $454 |
| Estimated incarcerated population (2008) [71] | 7,350 | 891,738 | 6,548 |
| GDP per capita (Purchasing Power Parity) (2009) (US$2009) [72] | $1,812 | $14,593 | $14,116 |

**References**

61. Toungoussova OS, Mariandyshev A, Bjune G, Sandven P, Caugant DA (2003) Molecular epidemiology and drug resistance of Mycobacterium tuberculosis isolates in the Archangel prison in Russia: predominance of the W-Beijing clone family. Clin Infect Dis 37: 665–672. doi:10.1086/377205.

67. Wares DF, Clowes CI (1997) Tuberculosis in Russia. Lancet 350: 957. doi:10.1016/S0140-6736(05)63295-3.

68. Zarbuev AN (2005) [The tuberculosis situation in the penitentiaries of Buryatia and measures of its stabilization]. Probl Tuberk Bolezn Legk: 13–16.

69. Ruddy M, Balabanova Y, Graham C, Fedorin I, Malomanova N, et al. (2005) Rates of drug resistance and risk factor analysis in civilian and prison patients with tuberculosis in Samara Region, Russia. Thorax 60: 130–135. doi:10.1136/thx.2004.026922.

70. Spradling P, Nemtsova E, Aptekar T, Shulgina M, Rybka L, et al. (2002) Anti-tuberculosis drug resistance in community and prison patients, Orel Oblast, Russian Federation. Int J Tuberc Lung Dis 6: 757–762.

71. Walmsley R (2009) World Prison Population List. London: International Centre for Prison Studies, King’s College London School of Law. p.

72. Central Intelligence Agency (2011) CIA - The World Factbook. Available:https://www.cia.gov/library/publications/the-world-factbook/. Accessed 21 June 2011.

73. Abubakar I, Story A, Lipman M, Bothamley G, van Hest R, et al. (2010) Diagnostic accuracy of digital chest radiography for pulmonary tuberculosis in a UK urban population. Eur Respir J 35: 689–692. doi:10.1183/09031936.00136609.
