# Supplementary material for: Screening and Rapid Molecular Diagnosis of Tuberculosis in Prisons in Russia and Eastern Europe: A Cost-Effectiveness Analysis
Source: PLoS Med. 2012 Nov 27;9(11):e1001348. doi: 10.1371/journal.pmed.1001348 (PMC3507963; doi:10.1371/journal.pmed.1001348)
Supplement: Table S3 — Results of univariate sensitivity analysis. (DOC) [file pmed.1001348.s007.doc]

| **Table S3.** Results of univariate sensitivity analysis. | | | |
| --- | --- | --- | --- |
| Parameter | Values | Cost-effectiveness frontier* | ICERs  ($/QALY) |
| Sensitivity of MMR for smear-positive cases | 0.587  0.688 | S6, S4  S6, S4 | $352  $814 |
| Sensitivity of MMR for smear-negative cases | 0.786  0.822 | S6, S4  S6, S4 | $539  $551 |
| Sensitivity of symptom screening for smear-positive cases | 0.542  0.627 | S6, S4  S6, S4 | $545  $545 |
| Sensitivity of symptom screening for smear-negative cases | 0.277  0.316 | S6, S4  S6, S4 | $545  $545 |
| Sensitivity of sputum PCR for smear-positive cases | 0.971  0.993 | S6, S4  S6, S4 | $577  $516 |
| Sensitivity of sputum PCR for smear-negative cases | 0.612  0.739 | S6, S4  S6, S4 | $567  $527 |
| Specificity of MMR for active TB | 0.980  0.982 | S6, S4  S6, S4 | $537  $554 |
| Specificity of symptom screening for active TB | 0.888  0.892 | S6, S4  S6, S4 | $545  $545 |
| Specificity of sputum PCR for active TB | 0.985  0.999 | S6, S4  S6, S4 | $605  $486 |
|  |  |  |  |
| Relative rate of re-infection among latently infected individuals (v) | 0.20  0.60 | S1, S2, S4  S6, S4 | $306; $1,798  $263 |
| Cost of sputum PCR | $18.06  $30.09 | S6, S4  S6, S5, S4 | $166  $750; $1,241 |
| Proportion of individuals rapidly progressing to active disease (q) | 0.128  0.213 | S6, S4  S6, S4 | $1,076  $244 |
| Contact rate for non-MDR TB (β_d_) | 5.25  8.75 | S6, S4  S6, S4 | $995  $225 |
| Prevalence of latent TB infection in the civilian population | 0.306  0.510 | S6, S4  S6, S4 | $970  $312 |
| Annual rate of rapid progression from latent to active TB (γ_2_) | 0.239  0.399 | S6, S4  S6, S4 | $948  $305 |
| Specificity of the clinical MDR detection process (DST + failure to progress on standard DOTS) | 0.743  1.00 | S6, S4  S2, S6, S5, S4 | $545  $371; $458; $703 |
| Cost of MDR-TB treatment | $5971  $9952 | S6, S4  S6, S4 | $693  $398 |
| Contact rate for MDR-TB | 5.25  8.75 | S2, S6, S4  S6, S4 | $398; $687  $392 |
| Annual death rate associated with untreated smear-positive non-MDR TB (ζ3_d_) | 0.158  0.263 | S6, S4  S6, S4 | $463  $631 |
| Cost of MMR | $3.64  $6.06 | S6, S4  S6, S4 | $623  $468 |
| Proportion of cases that are smear-positive (ρ) | 0.552  0.602 | S6, S4  S6, S4 | $622  $477 |
| Annual rate of self-presentation among smear-positive individuals between screening events | 0.129  0.225 | S2, S6, S4  S6, S4 | $10; $481  $613 |
| Baseline annual health expenditure | $156  $260 | S6, S4  S6, S4 | $482  $609 |
| Prevalence of a previous history of TB among the civilian population | 0  0.1 | S6, S4  S6, S4 | $545  $442 |
| Proportion of TB cases in the civilian population that are MDR | 0.0424  0.29 | S2, S4  S6, S4 | $541  $445 |
| Cost of treatment for smear-negative non-MDR TB | $273  $456 | S6, S4  S6, S4 | $596  $495 |
| Specificity of Xpert MTB/RIF for MDR-TB | 0.969  0.993 | S2, S6, S4  S6, S4 | $476; $532  $560 |
| Annual death rate associated with untreated smear-positive MDR-TB (ζ3_m_) | 0.158  0.263 | S6, S4  S6, S4 | $496  $592 |

| Parameter | Values | Cost-effectiveness frontier | ICERs  ($/QALY) |
| --- | --- | --- | --- |
| Annual rate of relapse to MDR-TB among those previously treated for non-MDR TB (z_2_) | 0.0264  0.111 | S6, S4  S6, S4 | $582  $512 |
| Cost of treatment for smear-positive non-MDR TB | $331  $551 | S6, S4  S6, S4 | $576  $515 |
| Annual rate of “self-cure” in which TB resolves without treatment (κ) | 0.0435  0.0725 | S6, S4  S6, S4 | $520  $572 |
| Annual rate of relapse to non-MDR TB among those previously treated for non-MDR TB (z_1_) | 0.0995  0.237 | S6, S4  S6, S4 | $571  $522 |
| Proportion of cases in the civilian population that are smear-positive | 0.23  0.66 | S6, S4  S6, S4 | $553  $528 |
| Annual rate of relapse to MDR-TB among those previously treated for MDR-TB (z_3_) | 0.327  1.321 | S6, S4  S6, S4 | $559  $534 |
| Specificity of the full diagnostic workup for active TB | 0.980  0.982 | S6, S4  S6, S4 | $536  $555 |
| Prevalence of active TB in the civilian population | 3.9 X 10^-4^  3.22 X 10^-3^ | S6, S4  S6, S4 | $548  $539 |
| Annual rate of slow progression from latent to active TB (γ_1_) | 4.9 X 10^-5^  8.16 X 10^-4^ | S6, S4  S6, S4 | $550  $540 |
| Annual death rate associated with untreated smear-negative MDR-TB (ζ2_m_) | 0.158  0.263 | S6, S4  S6, S4 | $550  $542 |
| Treatment success rate after treatment for MDR-TB (π_m_) | 0.475  0.557 | S6, S4  S6, S4 | $549  $542 |
| Annual rate at which MDR-TB develops through treatment-associated amplification | 0.0521  0.110 | S6, S4  S6, S4 | $548  $543 |
| Annual death rate associated with treatment for smear-positive MDR-TB | 0.0299  0.0471 | S6, S4  S6, S4 | $548  $543 |
| Annual rate of conversion from smear-negative to smear-positive (μ) | 0.0118  0.0197 | S6, S4  S6, S4 | $548  $543 |
| Coefficient of relative infectivity of smear-negative cases (D) | 0.20  0.28 | S6, S4  S6, S4 | $547  $543 |
| Treatment success rate after treatment for non-MDR TB (π_d_) | 0.738  2.01 | S6, S4  S6, S4 | $547  $544 |
| Annual death rate associated with treatment of smear-negative MDR-TB | 0.0299  0.0471 | S6, S4  S6, S4 | $547  $544 |
| Annual death rate from non-infectious causes in prisons of the FSU (ζ1) | 1.79 X 10^-3^  2.99 X 10^-3^ | S6, S4  S6, S4 | $544  $547 |
| Annual death rate associated with untreated smear-negative non-MDR-TB (ζ2_d_) | 0.0788  0.131 | S6, S4  S6, S4 | $547  $544 |
| Sensitivity of screening CXR for smear-positive TB on entry to the prison | 0.85  0.99 | S6, S4  S6, S4 | $545  $547 |
| Annual rate at which cases of acquired MDR-TB are detected and placed on DOTS-plus treatment in the absence of sputum PCR (ε_n_/ ε_p)_ | 7.72  12.9 | S6, S4  S6, S4 | $545  $546 |
| Annual death rate associated with treatment of smear-negative non-MDR TB | 2.39 X 10^-3^  1.55 X 10^-2^ | S6, S4  S6, S4 | $545  $546 |
| Annual rate of self-presentation among smear-negative cases between screening events | 0.129  0.225 | S6, S4  S6, S4 | $546  $549 |
| Treatment failure rate for non-MDR TB (τ_d_) | 0.0378  0.0815 | S6, S4  S6, S4 | $545  $546 |
| Death rate associated with treatment of smear-positive non-MDR TB | 2.39 X 10^-3^  1.55 X 10^-2^ | S6, S4  S6, S4 | $546  $545 |
| Cost of symptom screening | $1.64  $2.73 | S6, S4  S6, S4 | $546  $545 |
| Cost of sputum smear microscopy | $1.62  $2.70 | S6, S4  S6, S4 | $546  $545 |
| Proportion of smear-positive MDR-TB cases that would be placed on DOTS-plus treatment within one week if sputum PCR is used | 0.930  0.986 | S6, S4  S6, S4 | $545  $546 |

| Parameter | Values | Cost-effectiveness frontier | ICERs  ($/QALY) |
| --- | --- | --- | --- |
| Treatment failure rate for MDR-TB (τ_m_) | 0.148  0.186 | S6, S4  S6, S4 | $545  $546 |
| Annual rate at which smear-positive cases of MDR-TB misdiagnosed as DS-TB are started on DOTS-plus in the absence of sputum PCR | 3.86  6.43 | S6, S4  S6, S4 | $545  $546 |
| Starting prevalence of latent TB infection within the prison before the model is allowed to equilibrate | 0.6  1 | S6, S4  S6, S4 | $546  $545 |
| Starting prevalence of a previous history of TB among the prison population, before the model is allowed to equilibrate | 0  0.25 | S6, S4  S6, S4 | $545  $545 |
| Starting proportion of TB infection that is MDR in prison before model is allowed to equilibrate | 0.275  0.327 | S6, S4  S6, S4 | $545  $545 |
| Annual rate at which smear-positive cases of MDR-TB misdiagnosed as DS-TB are started on DOTS-plus in the absence of sputum PCR | 3.86  6.43 | S6, S4  S6, S4 | $545  $545 |
| Sensitivity of screening CXR for smear-negative TB on entry to the prison | 0.85  0.99 | S6, S4  S6, S4 | $545  $545 |
| Proportion of smear-negative MDR-TB cases that would be placed on DOTS-plus treatment within one week if sputum PCR is used | 0.592  0.724 | S6, S4  S6, S4 | $545  $545 |
| Starting prevalence of active TB infection within the prison before the model is allowed to equilibrate | 0.0216  0.036 | S6, S4  S6, S4 | $545  $545 |
| Starting fraction of active TB cases within the prison that are smear-positive before the model is allowed to equilibrate | 0.552  0.602 | S6, S4  S6, S4 | $545  $545 |
| Annual rate of self-presentation among smear-positive cases in the absence of screening (φ_p_) | 0.255  0.470 | S6, S4  S6, S4 | $545  $545 |
| Annual rate of self-presentation among smear-negative cases in the absence of screening (φ_n_) | 0.470  0.981 | S6, S4  S6, S4 | $545  $545 |

*** Strategies referred to by number in the table are as follows:**

**S1: Self-referral only (no screening)**

**S2: Annual MMR screening (status quo)**

**S3: Annual symptom screening**

**S4: Annual sputum PCR screening**

**S5: Annual combined MMR and symptom screening**

**S6: Annual MMR screening with sputum PCR for rapid MDR detection**

**S7: Annual symptom screening with sputum PCR for rapid MDR detection**

**S8: Annual combined MMR and symptom screening with sputum PCR for rapid MDR detection**

**Notably, “S6, S4”, the most common efficient frontier found in the table is the same as that of the main base case analysis of the study.**
