# Supplementary material for: Screening and Rapid Molecular Diagnosis of Tuberculosis in Prisons in Russia and Eastern Europe: A Cost-Effectiveness Analysis
Source: PLoS Med. 2012 Nov 27;9(11):e1001348. doi: 10.1371/journal.pmed.1001348 (PMC3507963; doi:10.1371/journal.pmed.1001348)
Supplement: Table S4 — Outcomes for selected alternative scenarios. (DOC) [file pmed.1001348.s008.doc]

| **Table S4.** Outcomes for selected alternative scenarios. | | | | | |
| --- | --- | --- | --- | --- | --- |
| Bacteriological cases are only true positive TB cases | Total Cost  ($2009) | Total QALYs | TB Prevalence (%) | MDR-TB Prevalence (%) | ICER ($/QALY) |
| **MMR screening (status quo)** | **$18,481,789** | **79,962** | **2.73** | **0.71** | **Reference** |
| MMR screening  with sputum PCR detection of MDR-TB | $18,486,064 | 79,976 | 2.70 | 0.66 | ~~$305~~* |
| Self-referral (no screening) | $18,519,768 | 79,733 | 4.01 | 0.94 | Dominated |
| **Sputum PCR screening** | **$18,526,871** | **80,117** | **2.15** | **0.59** | **$291** |
| Combined MMR and symptom screening | $18,578,380 | 80,064 | 2.30 | 0.64 | Dominated |
| Symptom screening | $18,606,840 | 79,913 | 3.06 | 0.73 | Dominated |
| Combined MMR and symptom screening  with sputum PCR detection of MDR-TB | $18,627,238 | 80,076 | 2.27 | 0.61 | Dominated |
| Symptom screening  with sputum PCR detection of MDR-TB | $18,647,845 | 79,928 | 3.02 | 0.68 | Dominated |
| Contact rate is 14 per year instead of 7 per year |  |  |  |  |  |
| **Sputum PCR screening** | **$18,321,181** | **80,382** | **1.70** | **0.51** | **Reference** |
| MMR screening  with sputum PCR detection of MDR-TB | $18,361,785 | 80,095 | 2.51 | 0.65 | Dominated |
| Combined MMR and symptom screening | $18,374,839 | 80,277 | 1.90 | 0.63 | Dominated |
| Combined MMR and symptom screening  with sputum PCR detection of MDR-TB | $18,379,982 | 80,311 | 1.84 | 0.54 | Dominated |
| MMR screening (status quo) | $18,411,578 | 80,047 | 2.60 | 0.79 | Dominated |
| Symptom screening  with sputum PCR detection of MDR-TB | $18,544,981 | 79,899 | 3.53 | 0.72 | Dominated |
| Symptom screening | $18,563,062 | 79,852 | 3.62 | 0.87 | Dominated |
| Self-referral (no screening) | $18,785,865 | 79,391 | 5.61 | 1.44 | Dominated |
| 60% of rapid-progressors develop disease within 1 year instead of 27% |  |  |  |  |  |
| **MMR screening**  **with sputum PCR detection of MDR-TB** | **$17,979,997** | **80,673** | **2.57** | **0.75** | **Reference** |
| MMR screening (status quo) | $17,989,551 | 80,654 | 2.61 | 0.81 | Dominated |
| **Sputum PCR screening** | **$18,031,609** | **80,809** | **1.96** | **0.61** | **$380** |
| Combined MMR and symptom screening | $18,033,547 | 80,761 | 2.09 | 0.69 | Dominated |
| Combined MMR and symptom screening  with sputum PCR detection of MDR-TB | $18,068,199 | 80,775 | 2.06 | 0.64 | Dominated |
| Symptom screening | $18,079,288 | 80,569 | 3.34 | 0.88 | Dominated |
| Symptom screening  with sputum PCR detection of MDR-TB | $18,100,523 | 80,588 | 3.31 | 0.81 | Dominated |
| Self-referral (no screening) | $18,115,984 | 80,358 | 4.71 | 1.25 | Dominated |
| 33% of individuals are rapid-progressors instead of 17% |  |  |  |  |  |
| **MMR screening**  **with sputum PCR detection of MDR-TB** | **$18,488,419** | **79,959** | **2.73** | **0.77** | **Reference** |
| MMR screening (status quo) | $18,498,411 | 79,940 | 2.77 | 0.83 | Dominated |
| **Sputum PCR screening** | **$18,501,065** | **80,154** | **2.20** | **0.64** | **$65** |
| Combined MMR and symptom screening | $18,514,014 | 80,088 | 2.31 | 0.72 | Dominated |
| Combined MMR and symptom screening  with sputum PCR detection of MDR-TB | $18,548,586 | 80,103 | 2.28 | 0.67 | Dominated |
| Symptom screening | $18,608,594 | 79,826 | 3.45 | 0.91 | Dominated |
| Symptom screening  with sputum PCR detection of MDR-TB | $18,628,312 | 79,845 | 3.40 | 0.84 | Dominated |
| Self-referral (no screening) | $18,672,177 | 79,580 | 4.49 | 0.124 | Dominated |

| **Table S4. Continued.** | | | | | |
| --- | --- | --- | --- | --- | --- |
| Without sputum PCR, only 50% of MDR cases are placed on DOTS-plus by 12 weeks of treatment | Total Cost  ($2009) | Total QALYs | TB Prevalence (%) | MDR-TB Prevalence (%) | ICER ($/QALY) |
| **MMR screening**  **with sputum PCR detection of MDR-TB** | **$18,527,440** | **79,884** | **2.75** | **0.69** | **Reference** |
| MMR screening (status quo) | $18,545,530 | 79,857 | 2.80 | 0.77 | Dominated |
| **Sputum PCR screening** | **$18,598,921** | **80,015** | **2.31** | **0.63** | **$546** |
| Combined MMR and symptom screening | $18,602,729 | 79,959 | 2.41 | 0.70 | Dominated |
| Symptom screening | $18,623,607 | 79,780 | 3.41 | 0.81 | Dominated |
| Combined MMR and symptom screening  with sputum PCR detection of MDR-TB | $18,630,684 | 79,981 | 2.37 | 0.64 | Dominated |
| Symptom screening  with sputum PCR detection of MDR-TB | $18,637,023 | 79,804 | 3.36 | 0.72 | Dominated |
| Self-referral (no screening) | $18,646,502 | 79,585 | 4.34 | 1.10 | Dominated |
| Without sputum PCR, 90% of MDR cases are placed on DOTS-plus by 16 weeks of treatment |  |  |  |  |  |
| **MMR screening (status quo)** | **$18,520,756** | **79,874** | **2.77** | **0.73** | **Reference** |
| **MMR screening**  **with sputum PCR detection of MDR-TB** | **$18,522,811** | **79,886** | **2.75** | **0.69** | **$171** |
| Self-referral (no screening) | $18,581,216 | 79,631 | 4.24 | 0.94 | Dominated |
| Combined MMR and symptom screening | $18,582,736 | 79,976 | 2.39 | 0.67 | ~~$666~~* |
| **Sputum PCR screening** | **$18,594,399** | **80,020** | **2.31** | **0.63** | **$534** |
| Symptom screening | $18,600,298 | 79,797 | 3.39 | 0.76 | Dominated |
| Combined MMR and symptom screening  with sputum PCR detection of MDR-TB | $18,626,126 | 79,984 | 2.37 | 0.64 | Dominated |
| Symptom screening  with sputum PCR detection of MDR-TB | $18,632,401 | 79,840 | 3.36 | 0.72 | Dominated |
| Without screening, 90% of smear-positives and 75% of smear-negatives will self-refer in a year |  |  |  |  |  |
| **MMR screening**  **with sputum PCR detection of MDR-TB** | **$18,524,341** | **79,886** | **2.75** | **0.69** | **Reference** |
| MMR screening (status quo) | $18,528,984 | 79,869 | 2.78 | 0.74 | Dominated |
| Self-referral (no screening) | $18,576,393 | 79,738 | 3.50 | 0.99 | Dominated |
| Combined MMR and symptom screening | $18,589,325 | 79,971 | 2.40 | 0.68 | ~~$764~~* |
| **Sputum PCR screening** | **$18,595,892** | **80,018** | **2.31** | **0.63** | **$542** |
| Symptom screening | $18,608,052 | 79,792 | 3.39 | 0.78 | Dominated |
| Combined MMR and symptom screening  with sputum PCR detection of MDR-TB | $18,627,632 | 79,984 | 2.37 | 0.64 | Dominated |
| Symptom screening  with sputum PCR detection of MDR-TB | $18,633,929 | 79,806 | 3.36 | 0.72 | Dominated |

| **Table S4. Continued.** |
| --- |

| Actual costs are 25% more than estimated in base case | Total Cost  ($2009) | Total QALYs | TB Prevalence (%) | MDR-TB Prevalence (%) | ICER ($/QALY) |
| --- | --- | --- | --- | --- | --- |
| **MMR screening**  **with sputum PCR detection of MDR-TB** | **$18,593,104** | **79,886** | **2.75** | **0.69** | **Reference** |
| MMR screening (status quo) | $18,595,518 | 79,869 | 2.78 | 0.74 | Dominated |
| Self-referral (no screening) | $18,650,873 | 79,614 | 4.28 | 0.99 | Dominated |
| Symptom screening | $18,683,069 | 79,792 | 3.39 | 0.78 | Dominated |
| Combined MMR and symptom screening | $18,686,579 | 79,971 | 2.40 | 0.68 | ~~$1,100~~* |
| **Sputum PCR screening** | **$18,702,721** | **80,018** | **2.31** | **0.63** | **$830** |
| Symptom screening  with sputum PCR detection of MDR-TB | $18,718,779 | 79,806 | 3.36 | 0.72 | Dominated |
| Combined MMR and symptom screening  with sputum PCR detection of MDR-TB | $18,737,042 | 79,984 | 2.37 | 0.64 | Dominated |
| Treatment for those released with active disease is delayed ^a^ |  |  |  |  |  |
| **MMR screening**  **with sputum PCR detection of MDR-TB** | **$18,504,936** | **75,821** | **2.75** | **0.69** | **Reference** |
| MMR screening (status quo) | $18,505,619 | 75,799 | 2.78 | 0.74 | Dominated |
| Combined MMR and symptom screening | $18,563,801 | 76,180 | 3.39 | 0.78 | ~~$164~~* |
| **Sputum PCR screening** | **$18,573,770** | **76,311** | **2.31** | **0.63** | **$140** |
| Self-referral (no screening) | $18,584,599 | 74,837 | 4.28 | 0.99 | Dominated |
| Symptom screening | $18,591,994 | 75,430 | 3.39 | 0.78 | Dominated |
| Combined MMR and symptom screening  with sputum PCR detection of MDR-TB | $18,605,259 | 76,197 | 2.37 | 0.64 | Dominated |
| Symptom screening  with sputum PCR detection of MDR-TB | $18,621,667 | 75,452 | 3.36 | 0.72 | Dominated |
| Screening occurs two times per year |  |  |  |  |  |
| **MMR screening (status quo)** | **$18,498,292** | **80,044** | **2.11** | **0.63** | **Reference** |
| **MMR screening**  **with sputum PCR detection of MDR-TB** | **$18,509,762** | **80,054** | **2.09** | **0.60** | **$1,069** |
| Self-referral (no screening) | $18,605,922 | 79,612 | 4.28 | 0.99 | Dominated |
| Symptom screening | $18,640,406 | 79,979 | 2.54 | 0.67 | Dominated |
| Combined MMR and symptom screening | $18,689,182 | 80,117 | 1.88 | 0.59 | ~~$2,847~~* |
| Symptom screening  with sputum PCR detection of MDR-TB | $18,712,985 | 79,991 | 2.52 | 0.64 | Dominated |
| **Sputum PCR screening** | **$18,749,173** | **80,146** | **1.84** | **0.56** | **$2,602** |
| Combined MMR and symptom screening  with sputum PCR detection of MDR-TB | $18,779,694 | 80,124 | 1.86 | 0.57 | Dominated |

| **Table S4. Continued.** | | | | | |
| --- | --- | --- | --- | --- | --- |
| Screening occurs every other year | Total Cost  ($2009) | Total QALYs | TB Prevalence (%) | MDR-TB Prevalence (%) | ICER ($/QALY) |
| **Sputum PCR screening** | **$18,574,006** | **79,826** | **3.42** | **0.71** | **Reference** |
| MMR screening  with sputum PCR detection of MDR-TB | $18,577,738 | 79,682 | 4.02 | 0.77 | Dominated |
| MMR screening (status quo) | $18,595,370 | 79,661 | 4.07 | 0.85 | Dominated |
| Combined MMR and symptom screening | $18,599,380 | 79,765 | 3.57 | 0.79 | Dominated |
| Self-referral (no screening) | $18,605,922 | 79,612 | 4.28 | 0.99 | Dominated |
| Combined MMR and symptom screening  with sputum PCR detection of MDR-TB | $18,606,330 | 79,784 | 3.53 | 0.73 | Dominated |
| Symptom screening  with sputum PCR detection of MDR-TB | $18,638,517 | 79,607 | 4.63 | 0.79 | Dominated |
| Symptom screening | $18,641,261 | 79,585 | 4.67 | 0.87 | Dominated |
| Drug shortage occurs for 6 weeks during 5^th^ year of model ^b^ |  |  |  |  |  |
| **MMR screening**  **with sputum PCR detection of MDR-TB** | **$18,526,293** | **79,882** | **2.75** | **0.69** | **Reference** |
| MMR screening (status quo) | $18,531,061 | 79,865 | 2.79 | 0.74 | Dominated |
| Combined MMR and symptom screening | $18,591,183 | 79,967 | 2.40 | 0.68 | ~~$763~~* |
| **Sputum PCR screening** | **$18,597,886** | **80,013** | **2.32** | **0.63** | **$547** |
| Self-referral (no screening) | $18,607,668 | 79,610 | 4.28 | 1.00 | Dominated |
| Symptom screening | $18,610,160 | 79,787 | 3.40 | 0.78 | Dominated |
| Combined MMR and symptom screening  with sputum PCR detection of MDR-TB | $18,629,587 | 79,979 | 2.37 | 0.64 | Dominated |
| Symptom screening  with sputum PCR detection of MDR-TB | $18,635,814 | 79,801 | 3.36 | 0.72 | Dominated |
| Mobile digital radiographic screening is used and has same cost as MMR ^c^ |  |  |  |  |  |
| MMR screening  with sputum PCR detection of MDR-TB | $18,491,589 | 79,945 | **2.54** | **0.66** | **Reference** |
| MMR screening (status quo) | $18,492,223 | 79,930 | 2.57 | 0.70 | Dominated |
| Combined MMR and symptom screening | $18,576,925 | 79,991 | 2.34 | 0.67 | ~~$1,855~~* |
| Sputum PCR screening | $18,595,892 | 80,018 | **2.31** | **0.63** | **$1,429** |
| Self-referral (no screening) | $18,604,958 | 79,614 | 4.28 | 0.99 | Dominated |
| Symptom screening | $18,608,052 | 79,792 | 3.39 | 0.78 | Dominated |
| Combined MMR and symptom screening  with sputum PCR detection of MDR-TB | $18,616,581 | 80,003 | 2.31 | 0.63 | Dominated |
| Symptom screening  with sputum PCR detection of MDR-TB | $18,633,929 | 79,806 | 3.36 | 0.72 | Dominated |

| Mobile digital radiographic screening is used and has 1/2 cost of MMR ^c^ | Total Cost  ($2009) | Total QALYs | TB Prevalence (%) | MDR-TB Prevalence (%) | ICER ($/QALY) |
| --- | --- | --- | --- | --- | --- |
| **MMR screening**  **with sputum PCR detection of MDR-TB** | **$18,470,742** | **79,945** | **2.54** | **0.66** | **Reference** |
| MMR screening (status quo) | $18,471,380 | 79,930 | 2.57 | 0.70 | Dominated |
| Combined MMR and symptom screening | $18,556,090 | 79,991 | 2.34 | 0.67 | ~~$1,855~~* |
| **Sputum PCR screening** | **$18,595,334** | **80,018** | **2.31** | **0.63** | **$1,707** |
| Combined MMR and symptom screening  with sputum PCR detection of MDR-TB | $18,595,742 | 80,003 | 2.31 | 0.63 | Dominated |
| Self-referral (no screening) | $18,604,659 | 79,614 | 4.28 | 0.99 | Dominated |
| Symptom screening | $18,605,490 | 79,792 | 3.39 | 0.78 | Dominated |
| Symptom screening  with sputum PCR detection of MDR-TB | $18,631,350 | 79,806 | 3.36 | 0.72 | Dominated |
| MMR has same sensitivity for smear-negative TB as for smear-positive TB |  |  |  |  |  |
| **MMR screening**  **with sputum PCR detection of MDR-TB** | **$18,529,451** | **79,865** | **2.88** | **0.70** | **Reference** |
| MMR screening (status quo) | $18,533,747 | 79,850 | 2.91 | 0.75 | Dominated |
| Combined MMR and symptom screening | $18,594,029 | 79,964 | 2.41 | 0.68 | ~~$652~~* |
| **Sputum PCR screening** | **$18,600,570** | **80,010** | **2.32** | **0.63** | **$490** |
| Self-referral (no screening) | $18,610,692 | 79,605 | 4.29 | 1.00 | Dominated |
| Symptom screening | $18,613,111 | 79,782 | 3.40 | 0.78 | Dominated |
| Combined MMR and symptom screening  with sputum PCR detection of MDR-TB | $18,632,396 | 79,976 | 2.38 | 0.64 | Dominated |
| Symptom screening  with sputum PCR detection of MDR-TB | $18,639,002 | 79,799 | 3.37 | 0.72 | Dominated |
| MMR is more sensitive for smear-positive TB as for smear-negative TB |  |  |  |  |  |
| **MMR screening**  **with sputum PCR detection of MDR-TB** | **$18,495,624** | **79,855** | **2.81** | **0.68** | **Reference** |
| MMR screening (status quo) | $18,498,272 | 79,840 | 2.84 | 0.73 | Dominated |
| Combined MMR and symptom screening | $18,568,898 | 79,930 | 2.45 | 0.68 | ~~$974~~* |
| **Sputum PCR screening** | **$18,580,782** | **79,978** | **2.31** | **0.63** | **$688** |
| Self-referral (no screening) | $18,588,216 | 79,578 | 4.27 | 0.99 | Dominated |
| Symptom screening | $18,592,034 | 79,753 | 3.39 | 0.77 | Dominated |
| Combined MMR and symptom screening  with sputum PCR detection of MDR-TB | $18,607,882 | 79,942 | 2.43 | 0.64 | Dominated |
| Symptom screening  with sputum PCR detection of MDR-TB | $18,618,072 | 79,770 | 3.36 | 0.72 | Dominated |
| Individuals recovered from active MDR-TB can be reinfected with both MDR-TB and non-MDR-TB with treatment of subsequent active disease being first-line for non-MDR and second-line for MDR |  |  |  |  |  |
| **MMR screening**  **with sputum PCR detection of MDR-TB** | **$18,522,204** | **79,887** | **2.88** | **0.70** | **Reference** |
| MMR screening (status quo) | $18,526,813 | 79,870 | 2.91 | 0.75 | Dominated |
| Combined MMR and symptom screening | $18,587,508 | 79,971 | 2.41 | 0.68 | ~~$777~~* |
| **Sputum PCR screening** | **$18,594,540** | **80,018** | **2.32** | **0.63** | **$552** |
| Symptom screening | $18,605,813 | 79,791 | 3.40 | 0.78 | Dominated |
| Combined MMR and symptom screening  with sputum PCR detection of MDR-TB | $18,626,002 | 79,984 | 2.38 | 0.64 | Dominated |
| Symptom screening  with sputum PCR detection of MDR-TB | $18,631,636 | 79,807 | 3.37 | 0.72 | Dominated |
| Self-referral (no screening) | $18,755,076 | 79,180 | 4.29 | 1.00 | Dominated |

*Dominated by extended dominance.

^a^ Treatment delay was modeled via worse treatment outcomes for the first 6 months after release: treatment failure and death are 3 times more likely; acquired MDR is 4/3 as likely.

^b^ A drug shortage was modeled by inserting a period of 6 (non-screening) weeks during which individuals with non-MDR TB under standard DOTS therapy are 5 times more likely to acquire MDR-TB and 5 times more likely to fail treatment.

^c^ Mobile digital radiography was modeled to have a sensitivity of 0.77 for both smear-positive and smear-negative TB [73].

**References**

73. Abubakar I, Story A, Lipman M, Bothamley G, van Hest R, et al. (2010) Diagnostic accuracy of digital chest radiography for pulmonary tuberculosis in a UK urban population. Eur Respir J 35: 689–692. doi:10.1183/09031936.00136609.
