# Supplementary material for: Screening and Rapid Molecular Diagnosis of Tuberculosis in Prisons in Russia and Eastern Europe: A Cost-Effectiveness Analysis
Source: PLoS Med. 2012 Nov 27;9(11):e1001348. doi: 10.1371/journal.pmed.1001348 (PMC3507963; doi:10.1371/journal.pmed.1001348)
Supplement: Table S5 — Health states and their transitions. (DOC) [file pmed.1001348.s009.doc]

| **Table S5. Health States and their transitions** | |
| --- | --- |
| Health state | Equation defining transitions into and out of health state |
| Susceptible | $\frac{dS}{dt}=b_{1}-\lambda_{d}(1-q)S-\lambda_{d}qS-\lambda_{m}(1-q)S-\lambda_{m}qS-\zeta_{1}$ |
| Latent infection, non-MDR, slow progressing | $\frac{dL_{ds}}{dt}=b_{2d}+\lambda_{d}(1-q)S+v\lambda_{d}L_{ms}(1-q)-\gamma_{1}L_{ds}(1-\rho)-\gamma_{1}L_{ds}\rho-v\lambda_{d}L_{ds}-v\lambda_{m}L_{ds}(1-q)-v\lambda_{m}L_{ds}q-\zeta_{1}$ |
| Latent infection, non-MDR, fast progressing | $\frac{dL_{df}}{dt}=b_{3d}+\lambda_{d}qS+v\lambda_{d}L_{ds}+v\lambda_{d}L_{ms}q+(z_{1}+v\lambda_{d})R_{d}-\gamma_{2}L_{df}(1-\rho)-\gamma_{2}L_{df}\rho-\zeta_{1}$ |
| Latent infection, MDR, slow progressing | $\frac{dL_{ms}}{dt}=b_{2m}+\lambda_{m}(1-q)S+v\lambda_{m}L_{ds}(1-q)-\gamma_{1}L_{ms}(1-\chi)-\gamma_{1}L_{ms}\chi-v\lambda_{m}L_{ms}-v\lambda_{d}L_{ms}(1-q)-v\lambda_{d}L_{ms}q-\zeta_{1}$ |
| Latent infection, MDR, fast progressing | $\frac{dL_{mf}}{dt}=b_{3m}+\lambda_{m}qS+v\lambda_{m}L_{ms}+v\lambda_{m}L_{ds}+(z_{2}+v\lambda_{m})R_{d}+(z_{3}+v\lambda_{m})R_{m}-\gamma_{2}L_{mf}(1-\chi)-\gamma_{2}L_{mf}\chi-\zeta_{1}$ |
| Active disease, non-MDR, smear-negative, undetected |  |
| Active disease, non-MDR, smear-positive, undetected |  |
| Active disease, non-MDR, smear-negative, DOTS |  |
| Active disease, non-MDR, smear-positive, DOTS | $\frac{dA_{dpt}}{dt}=b_{7d}+\phi_{p}A_{dp}+\phi'_{p}A_{dp}-\pi_{d}A_{dpt}-\tau_{d}A_{dpt}-\sigma A_{dpt}-\zeta_{5d}$ |
| Active disease, MDR,  smear-negative, undetected |  |
| Active disease, MDR,  smear-positive, undetected |  |
| Active disease, MDR,  smear-negative, DOTS |  |
| Active disease, MDR,  smear-positive, DOTS |  |
| Active disease, MDR,  smear-negative, DOTS-plus | $\frac{dA_{mnt}}{dt}=b_{6m}+\varepsilon_{n}A_{mnx}+\psi A_{mna}-\pi_{m}A_{mnt}-\tau_{m}A_{mnt}-\zeta_{4m}$ |
| Active disease, MDR,  smear-positive, DOTS-plus | $\frac{dA_{mpt}}{dt}=b_{7m}+\varepsilon_{p}A_{mpx}+\psi A_{mpa}-\pi_{m}A_{mpt}-\tau_{m}A_{mpt}-\zeta_{5m}$ |
| Acquired MDR, smear-negative, DOTS | $\frac{dA_{mna}}{dt}=b_{11m}+FA_{dnt}+\nu\lambda_{m}A_{dnt}-\mu A_{mna}-\psi A_{mna}-\kappa A_{mna}-\xi_{2m}$ |
| Acquired MDR, smear-positive, DOTS | $\frac{dA_{mpa}}{dt}=b_{11m}+\sigma A_{dpt}+\mu A_{mna}-\psi A_{mpa}-\kappa A_{mpa}-\xi_{2m}$ |
| Recovered from non-MDR disease | $\frac{dR_{d}}{dt}=b_{8d}+\pi_{d}A_{dnt}+\pi_{d}A_{dpt}+\kappa A_{dn}+\kappa A{}_{dp}-(z_{1}+v\lambda_{d})R_{d}-(z_{2}+v\lambda_{m})R_{d}-\zeta_{1}$ |
| Recovered from MDR disease | $\frac{dR_{m}}{dt}=b_{8m}+\pi_{m}(A_{mnt}+A_{mpt})+\kappa(A_{mn}+A_{mp}+A_{mnx}+A_{mpx}+A_{mna}+A_{mpa})-(z_{3}+v\lambda_{m})R_{m}-\zeta_{1}$ |
| Chronic disease | $\frac{dCh}{dt}=b_{9}+\tau_{d}A_{dnt}+\tau_{d}A_{dpt}+\tau_{m}A_{mnt}+\tau_{m}A_{mpt}-\zeta_{3d}$ |
| Force of infection for drug-sensitive TB | $\lambda_{d}=\frac{(\beta_{d}A_{dp}+D\beta_{d}A_{dn})}{N-(A_{dpt}+A_{mpt}+A_{mnt}+A_{mpx}+A_{mpa})}$ |
| Force of infection for MDR-TB | $\lambda_{m}=\frac{(\beta_{m}A_{mp}+D\beta_{m}A_{mn}+D\beta_{m}A_{mnx}+D\beta_{m}A_{mna})}{N-(A_{dpt}+A_{mpt}+A_{mnt}+A_{mpx}+A_{mpa})}$ |
| Rate of treatment amplification and re-infection with MDR | $\sigma=F+\frac{\nu(\beta_{m}A_{mpx}+\beta_{m}A_{mpa})}{(A_{dpt}+A_{mpx}+A_{mpa})}$ |
