# Supplementary material for: Screening and Rapid Molecular Diagnosis of Tuberculosis in Prisons in Russia and Eastern Europe: A Cost-Effectiveness Analysis
Source: PLoS Med. 2012 Nov 27;9(11):e1001348. doi: 10.1371/journal.pmed.1001348 (PMC3507963; doi:10.1371/journal.pmed.1001348)
Supplement: Table S6 — Data used to estimate test characteristics of MMR and symptom screening. (DOC) [file pmed.1001348.s010.doc]

| **Table S6.** Data used to estimate test characteristics of MMR and symptom screening | | | | | | | | | |
| --- | --- | --- | --- | --- | --- | --- | --- | --- | --- |
|  |  | Bacteriological cases only | | | | 46.7% of “abacillary cases” are included | | | |
| Study | Disease form | MMR (+) | MMR  (–) | Symptom screen (+) | Symptom screen  (–) | MMR (+) | MMR  (–) | Symptom screen (+) | Symptom screen  (–) |
| Datta et al. [36] | Smear-positive | 38 | 38 | 61 | 15 | 38 | 38 | 61 | 15 |
|  | Smear-negative | 29 | 21 | 35 | 15 | 191 | 21 | 87 | 141 |
|  | No disease | 346 | 15545 | 5532 | 10359 | 184 | 15545 | 5355 | 10359 |
| Gopi et al. [37] | Smear-positive | 38 | 38 | 178 | 102 | 235 | 44 | 178 | 102 |
|  | Smear-negative | 29 | 21 | 101 | 135 | 1733 | 43 | 590 | 1186 |
|  | No disease | 346 | 15545 | 7347 | 80970 | 1758 | 83199 | 5807 | 80970 |
| Lewis et al. [38] | Smear-positive | 5 | 8 | 4 | 9 | 5 | 8 | 4 | 9 |
|  | Smear-negative | 8 | 30 | 11 | 27 | 20 | 30 | 15 | 35 |
|  | No disease | 25 | 1877 | 184 | 1718 | 13 | 1877 | 172 | 1718 |
| den Boon et al. | Smear-positive | – | – | 15 | 5 | – | – | 15 | 5 |
| [40] | Smear-negative | – | – | 5 | 4 | – | – | 34 | 66 |
|  | No disease | – | – | 369 | 771 | – | – | 278 | 771 |
| Churchyard et al. | Smear-positive | – | – | 95 | 55 | – | – | 95 | 55 |
| [39] | Smear-negative | – | – | 71 | 99 | – | – | 71 | 99 |
|  | No disease | – | – | NA | NA | – | – | NA | NA |
| Total | Smear-positive | 278 | 90 | 353 | 186 | 278 | 90 | 353 | 186 |
|  | Smear-negative | 230 | 94 | 223 | 280 | 1943 | 94 | 796 | 1527 |
|  | No disease | 3669 | 101478 | 13432 | 93818 | 1956 | 100621 | 11612 | 93818 |
| Pooled sensitivity | Smear-positive |  | 0.755 |  | 0.655 |  | 0.755 |  | 0.655 |
|  | Smear-negative |  | 0.710 |  | 0.443 |  | 0.954 |  | 0.343 |
| Adjusted sensitivity* | Smear-positive |  | **0.641** |  | **0.586** |  | **0.638** |  | **0.585** |
|  | Smear-negative |  | **0.627** |  | **0.402** |  | **0.804** |  | **0.296** |
| Specificity |  |  | **0.965** |  | **0.875** |  | **0.981** |  | **0.890** |

* Pooled sensitivity estimates were adjusted for verification bias by correcting individual sensitivity estimates of studies in which no data was collected regarding false negatives of initial combined radiographic and symptom screen [36,37] before pooling. These individual estimates were corrected by 0.841, the sensitivity of the combined screen in the two studies about which such data were reported [38,40].
