# Supplementary material for: Screening and Rapid Molecular Diagnosis of Tuberculosis in Prisons in Russia and Eastern Europe: A Cost-Effectiveness Analysis
Source: PLoS Med. 2012 Nov 27;9(11):e1001348. doi: 10.1371/journal.pmed.1001348 (PMC3507963; doi:10.1371/journal.pmed.1001348)
Supplement: Table S7 — Cost components. (DOC) [file pmed.1001348.s011.doc]

| **Table S7.** Cost components | |  | |  | | | |
| --- | --- | --- | --- | --- | --- | --- | --- |
| Item | Indirect costs* | | Capital costs | | Material costs | Labor costs | Unit cost |
| Mass miniature radiography | 0.65 | | 0.86 | | 0.38 | 2.96 | $4.85 |
| Symptom screening | 0.85 | | – | | – | 1.34 | $2.19 |
| Sputum smear | 0.59 | | 0.02 | | 1.09 | 0.47 | $2.16 |
| PCR | – | | – | | – | – | $24.08^a^ |
| Treatment of smear-negative non-MDR TB | 5.54 | | 4.58 | | 329.83 | 24.51 | $364.45 |
| Treatment of smear-positive non-MDR TB | 21.64 | | 17.13 | | 329.83 | 72.81 | $441.42 |
| Treatment of MDR-TB | – | | – | | – | – | $7,961.02† |

* Indirect costs include the costs of overhead, supervision, training and quality control.

^a^ Costs for sputum PCR adjusted from [42]. Reported cost components were segregated into tradable and non-tradable costs. Non-tradable costs were then projected by scaling based on the ratios of the logarithms of per-capita GDP of the countries included in [42] in relationship to the differences in costs in these countries, while tradable costs were held constant.

†The cost of treating MDR-TB was estimated from the reported whole budget of a pilot program for establishing treatment for 50 MDR-TB patients in the civilian sector of Tajikistan. Individual ingredients were not available to us.
