# Supplementary material for: Screening and Rapid Molecular Diagnosis of Tuberculosis in Prisons in Russia and Eastern Europe: A Cost-Effectiveness Analysis
Source: PLoS Med. 2012 Nov 27;9(11):e1001348. doi: 10.1371/journal.pmed.1001348 (PMC3507963; doi:10.1371/journal.pmed.1001348)
Supplement: Table S8 — Health state utility weights. (DOC) [file pmed.1001348.s012.doc]

| **Table S8.** Health state utility weights [14,15] | |
| --- | --- |
| Health state | Utility weight* |
| Susceptible | 1.00 |
| Latent infection, non-MDR, slow progressing | 1.00 |
| Latent infection, non-MDR, fast progressing | 1.00 |
| Latent infection, MDR, slow progressing | 1.00 |
| Latent infection, MDR, fast progressing | 1.00 |
| Active disease, non-MDR, smear-negative, undetected | 0.73 |
| Active disease, non-MDR, smear-positive, undetected | 0.73 |
| Active disease, non-MDR, smear-negative, DOTS | 0.68 |
| Active disease, non-MDR, smear-positive, DOTS | 0.60 |
| Active disease, MDR, smear-negative, undetected | 0.73 |
| Active disease, MDR, smear-positive, undetected | 0.73 |
| Active disease, MDR, smear-negative, DOTS-plus | 0.60 |
| Active disease, MDR, smear-positive, DOTS-plus | 0.60 |
| Active disease, MDR, smear-negative, DOTS | 0.68 |
| Active disease, MDR, smear-positive, DOTS | 0.60 |
| Acquired MDR, smear-negative, DOTS | 0.68 |
| Acquired MDR, smear-positive, DOTS | 0.60 |
| Recovered from non-MDR disease | 1.00 |
| Recovered from MDR disease | 1.00 |
| Chronic disease | 0.35 |

* While some studies have shown increases in health state utilities while on treatment, studies [14, 15] show decrements consistent with the observation that, unlike the general population, in the FSU prison population, for many prisoners forced quarantine or semi-isolation is the norm while on treatment. Furthermore, prisoners known to have active TB are often strongly stigmatized and shunned by fellow inmates.
