# Supplementary material for: Screening and Rapid Molecular Diagnosis of Tuberculosis in Prisons in Russia and Eastern Europe: A Cost-Effectiveness Analysis
Source: PLoS Med. 2012 Nov 27;9(11):e1001348. doi: 10.1371/journal.pmed.1001348 (PMC3507963; doi:10.1371/journal.pmed.1001348)
Supplement: Text S1 — Technical appendix. (DOC) [file pmed.1001348.s013.doc]

**Text S1: Technical Appendix**

*Analytic Overview*

We developed a dynamic state-transition model to estimate the incremental cost and quality-adjusted life years (QALYs) gained under different strategies for TB case finding in prisons of the former Soviet Union (FSU), where multi-drug resistant TB (MDR-TB) is highly prevalent. Our model allows prevalent drug-susceptible and MDR-TB cases to influence the incidence of new infections, enabling it to capture dynamic effects not permitted in cohort Markov models. For the main analysis, model inputs were selected to simulate a TB epidemic in an average FSU prison. Taking the health system perspective, we consider costs accrued from screening, diagnostic testing, treatment of TB and MDR-TB and baseline health expenditure, both within the prison and after inmates are released. Our analyses were conducted over a 10 year time span where the health benefits and costs of screening inmates during that time period were considered along with the remaining life time health benefits and costs of these inmates even beyond the end of the 10 year period.

*Model structure*

Our TB transmission model is a deterministic compartmental model composed of 16 different health-states (Figure S1, Table S1 and Table S5). A system of ordinary differential equations defines the transition rates of individuals between these states (Table S5). We assumed density-dependent homogeneous mixing. Given that many FSU prisons have barrack-style housing with up to 80 inmates living in one enclosed space, this assumption may be more realistic than in other settings where TB has been modeled. We developed our model in Matlab 7.12 (MathWorks).

Individuals enter the model through arrest and sentencing, and leave the model either through death or release. The relative proportion of entries into each health state is determined on the basis of reported country-wide epidemiological data, taking into account radiographic screening on entry. These proportions remain fixed through the time horizon of the model. Death and release can occur from any health state. Release is assumed to occur at an equal rate in all health states. In all analyses, 33% of inmates are released each year [7]. Costs and QALYs after release were projected using Markov models, described further below. Death from non-TB causes occurs at a baseline rate of 233 per 100,000 per year [17], while in active disease states, death rates reflect published rates for treated and untreated disease.

Upon infection, susceptible individuals enter latent infection states that are specific to the susceptibility profile of the TB strain with which they have been infected. Non-MDR infection is modeled to be 18% mono-resistant (with 7.7% mono-resistance to isoniazid) and 25% poly-resistant [23,43]. Latent infection is divided into slow- and fast-progressing health states, reflecting differences in inherent host characteristics [7,46,47]. Re-infection can occur from slow-progressing latent states, with an assumed degree of partial immunity (v = 0.48) [48,49]. Re-infection can occur with either non-MDR or MDR-TB, regardless of the drug-susceptibility profile of the TB strain with which an individual is latently infected. The proportion of new infections and re-infections progressing rapidly to active disease was assumed to be at the upper end of the reported range of values (q = 0.17), given the poor baseline health status and the low but rising prevalence of HIV-infection among FSU prisoners [7,46,47].

Latently infected individuals remain non-infectious, until they progress to active disease states. Active disease is divided into smear-negative and smear-positive TB, the latter representing cases detectable by direct sputum smear microscopy. Undetected cases of active disease, both smear-negative and smear-positive, are infectious, and therefore contribute to the force of infection (λ) for a given strain of TB (Table S5). Smear-negative cases contribute less to the force of infection (i.e. are less infectious) than smear-positive disease by a coefficient (D) [50–52].

After treatment, individuals enter either strain-specific recovered states or the chronic TB disease state. In this state, individuals are assumed to be provided with “suppressive” therapy and/or are isolated, such that they are not infectious. Individuals recovering from non-MDR disease can have recurrent disease either through relapse or re-infection with either non-MDR or MDR-TB strains. Individuals recovering from MDR disease can have recurrent disease either through relapse or re-infection only with MDR-TB strains. The tendency of the model toward an increased proportion of MDR-TB cases was counteracted by the constant proportion of non-MDR TB infection among individuals entering the prison.

HIV-infection is not explicitly considered, either in the main transmission model or in the QALY/life-expectancy calculations as accurate and consistent estimates of HIV prevalence for the prisons of the FSU along with accurate, FSU-specific HIV-related life expectancy reductions in these prison setting are not available.

*Model calibration*

Under the current case detection strategy—MMR screening followed by smear microscopy—TB transmission was allowed to reach a steady-state equilibrium before the strategy was continued or other interventions were implemented. No time series data could be identified with which to calibrate our model. However, in order to fit our model to the epidemiological situations of the countries for which we performed analyses, we manually varied the prevalence of latent infection in the general population within observed ranges to match the prevalence of active disease within prisons to observed rates in published data (PrL = 0.53 [0.23-0.63]). This is in keeping methodologically with consensus guidelines for assessing morbidity of tuberculosis within a large population [53]. However, the range of latent disease prevalence within which we varied our model parameter was derived from empirical observation among a high-risk population in the Russian Federation (health care workers) [54].

*Screening and diagnosis*

Individuals with active disease remain infectious until they are detected either through self-referral or screening. We evaluated 7 strategies for screening as well as the strategy of self-referral only. Screening was modeled as a discrete annual event. Self-referral occurs at a constant rate (ϕ_n/p_ = 0.1761) in all strategies with active case finding [25]. In the strategy of self-referral only (no screening), it was assumed that 60% of smear-positive and 30% of smear-negative individuals would present for care within a year. Screening methods evaluated were symptom questionnaires, MMR, both MMR and symptom screening combined and Xpert MTB/RIF alone. The diagnostic evaluation for all individuals who screen positive includes symptom history, MMR and direct sputum smear microscopy.

The sensitivity and specificity of each screening/diagnosis combination determines the proportion of individuals with undetected disease who are ultimately detected and treated (Table 1). Sensitivities and specificities were estimated separately for smear-positive and smear negative disease. Estimates for different testing methods were combined into individual case identification strategies with conditional independence assumed. Sensitivity estimates were drawn from data reported in field-based prevalence surveys of active TB [42,37,41,55]. These studies were chosen because of their use of a clinical case definition of active pulmonary TB.

Because little data on the performance characteristics of MMR was readily available, we conducted a systematic review of the literature to establish the most accurate possible parameter estimates. We searched in MEDLINE, using the following search terms: “mass” + ”miniature” + ”radiography”; “chest” + ”photofluorography”; “chest” + “photofluoroscopy”; “photofluorographic” + ”screening” + ”tuberculosis”; “abreugraphy.” From the results of these search terms, 281 original titles were identified and screened for relevance. From among these titles, 52 article abstracts were reviewed for relevance, 9 of which were downloaded in their full text. An additional 37 potentially relevant articles did not have abstracts available online and were reviewed in their original form in scanned or hardcopy archived journals. Articles were excluded if they were in languages other than English, Russian or Spanish, if bacteriological confirmation of cases was not used, if data could not be disaggregated to provide separate estimates for smear-positive and smear-negative disease, or if MMR was used as the only method for identifying TB suspects. In addition, we examined article references identified as potentially relevant in this search. Using this method, 3 articles were identified for use in developing parameter estimates [37–439].

In developing our parameter estimates, we separately examined the sensitivity for bacteriologically positive cases of TB (cases that are smear-positive, culture-positive or both) and “abacillary cases” of pulmonary TB (i.e. cases with radiographic and clinical evidence of TB disease, but who could either produce no sputum, or who were culture negative) (Table S6). As noted, in studies using more invasive techniques (e.g. bronchoalveolar lavage) to establish a bacteriological diagnosis in these cases, approximately 46.7% of “abacillary cases” can be confirmed to be tuberculosis [31–34]. Therefore, in our base case estimate of the sensitivity and specificity of MMR, 46.7% of “abacillary cases” were assumed to be true positives, and 53.3% assumed to be false positives. In addition, two studies were reviewed, in which cases missed by combined radiographic and symptom screening were identified either by concurrent bacteriological sputum examination or by 3-month follow up of the entire study population. In these studies, the sensitivity of combined screening was 84.1% and the specificity was 89.9% [39,41]. We used these estimates to adjust for verification bias in our calculations of the performance characteristics of individual screening components.

For mass miniature radiography, screening was assumed to be positive if any TB-related abnormality was seen on the reading of the radiograph. Among 692 bacteriologically positive TB cases in the studies we reviewed (adjusted for verification bias), MMR had a sensitivity of 64.1% for smear-positive disease and 62.7% for smear-negative disease, and a specificity of 96.5%. Assuming 46.7% of the 1,713 “abacillary cases” were true positives, the sensitivity for smear-positive disease is 63.7%, the sensitivity for smear-negative disease is 80.4% and the specificity is 98.1% [37–39]. Because of the assumptions needed between studies regarding correction for verification bias, in sensitivity analyses we assumed that MMR’s sensitivity for smear-negative cases was equal to that of its sensitivity for smear-positive cases. Results of this sensitivity analysis are shown in Table S4 and do not differ substantially in terms of health benefits, strategy ranking, or incremental cost-effectiveness from those of the main analysis.

Data regarding the sensitivity of symptom screening for pulmonary TB was more readily available than for MMR. Symptom screening was considered positive if any cardinal TB symptom was present. Where possible, the proportion of “abacillary cases” assumed to be true positives that exhibited symptoms was determined. Among 1,042 bacteriologically positive TB cases in the studies we reviewed (adjusted for verification bias), symptom screening had a sensitivity of 58.6% for smear-positive disease and 40.2% for smear-negative disease, and a specificity of 87.5%. Assuming 46.7% of the 1,820 “abacillary cases” were true positives, the sensitivity for smear-positive disease is 58.5%, the sensitivity for smear-negative disease is 29.6% and the specificity is 89.0% [37–41].

We estimated the sensitivity (84.9% for smear-positive disease and 86.2% for smear-negative disease) and specificity (87.3%) of combined MMR and symptom screening, assuming conditional independence. Data regarding cases missed by combined radiographic and symptom screening in the studies mentioned above could not be disaggregated by smear-positivity; however, the sensitivity and specificity (84.1% and 90.0% respectively) of combined screening, as directly measured in those studies, was comparable [39,41].

The sensitivity and specificity estimates of sputum PCR for smear-positive and smear-negative disease were drawn from a recent multi-center trial of the Xpert MTB/RIF conducted by Boehme et al. [42]. This is a recently-developed, commercially-available, automated system, which uses RT-PCR with probes for in strategies using rapid sputum PCR – detection of DNA specific to *Mycobacterium* tuberculosis and rifampicin resistance with Xpert MTB/RIF. In this multi-national, multi-center trial with 1730 patients, the sensitivity of Xpert MTB/RIF was 98.2% and 72.5% for smear-positive and smear-negative disease, respectively. The specificity of Xpert MTB/RIF for culture-positive TB was 99.2%. In addition, this study provided data regarding 105 “abacillary cases” of TB diagnosed clinically. Of these cases, 25 were found to be positive for the presence of M. *tuberculosis* on sputum PCR using Xpert MTB/RIF. Assuming that 46.7% of these “abacillary cases” are true positives and the above-mentioned specificity, Xpert MTB/RIF was estimated to be 67.5% sensitive for smear-negative disease. Because the sensitivity of Xpert MTB/RIF did not need to be adjusted for verification bias, the estimated sensitivity for smear-positive disease and specificity remained the same [42].

In our model, the estimated performance characteristics of Xpert MTB/RIF could impact MDR-TB detection and transmission in two ways. First, when used as a stand-alone screening tool, the sensitivity of Xpert MTB/RIF for smear-positive and smear-negative TB disease directly determine the rates of transition from all undetected active disease states to detected/treated states, thus impacting the rate at which all infectious individuals are isolated. Second, in all strategies including the use of sputum PCR, whether as a screening tool or as a form of rapid DST for MDR detection, the sensitivity and specificity of Xpert MTB/RIF for MDR-TB determines the rate at which MDR-TB cases transition from standard first-line (DOTS) therapy to second-line (DOTS-plus) regimens (see below).

*Treatment*

Cases of active non-MDR disease detected via case finding activities or self-referral enter one of two treatment states: smear-positive non-MDR TB on first-line treatment or smear-negative non-MDR TB on first-line treatment. Individuals in the non-MDR TB treatment states are treated for six months with a standard four-drug regimen in accordance with the WHO’s Directly Observed Therapy Short-course strategy (DOTS) [56]. Individuals with smear-positive disease are hospitalized during the 2-month intensive phase of therapy, whereas smear-negative cases are treated entirely on an outpatient basis. If a diagnosis of TB is confirmed, sputum culture and drug susceptibility testing are performed, in order to determine the drug-susceptibility profile of the infected individual’s TB strain.

Cases of active MDR disease detected via case finding activities or self-referral first enter one of two initial treatment states: smear-positive MDR-TB on first-line therapy or smear-negative MDR-TB on first-line therapy. In these states, individuals with MDR-TB are treated empirically with standard first-line therapy before the multidrug resistance of their strain is detected. Treatment with second-line therapy is initiated on the basis of drug susceptibility testing, failure to improve or convert to sputum negativity after a second intensive phase of treatment with DOTS (category II), or – in strategies using rapid PCR – detection of DNA specific to *Mycobacterium* tuberculosis and rifampicin resistance the rpoB gene with Xpert MTB/RIF, transition from initial first-line therapy to second-line treatment state is assumed to occur within 14 weeks (approximating the time to result for DST) of case detection for 75% of individuals. In strategies including the use of Xpert MTB/RIF, rates of transition from first-line therapy to second-line therapy depend on the sensitivity of the test for tuberculosis (98.2% for smear-positive cases and 67.5% for smear-negative cases), and on the sensitivity for multi-drug resistance (97.5%) [42]. Therefore, the probability of detecting multidrug resistance, conditional on detecting *Mycobacterium* tuberculosis with Xpert MTB/RIF, was 0.958 for smear-positive MDR-TB and 0.658 for smear-negative MDR-TB cases. Individuals detected in this way are placed on second-line therapy within 1 week of being diagnosed with TB. Individuals diagnosed with MDR-TB are treated on second-line therapy for 24 months (administered entirely as an inpatient) with ≥ 5 drugs in accordance with DOTS-plus [9].

Studies of DOTS-based treatment of non-MDR TB, in which the acquisition MDR-TB was reported, were used to estimate treatment outcomes in our model for first-line therapy [23,44]. Treatment of MDR-TB using second-line therapy was matched to outcomes reported from studies of DOTS-plus in FSU settings [9,29,44,45]. In our model, acquisition of MDR-TB strains during DOTS treatment has two components: a static component (F) associated with amplification of existing resistance, and a dynamic component associated with re-infection. Existing genotyping studies of acquired resistance were analyzed to determine the static rate of treatment-associated amplification of resistance [43,44].

Treatment-acquired drug-resistance was modeled as a transition from non-MDR TB treatment states first to “intermediate” treatment states in which the acquisition of resistance has yet to be discovered, then subsequently to second-line treatment. In these “intermediate” states, smear-positive and smear-negative individuals accrue treatment costs associated with first-line treatment, but can contribute to the local force of infection for MDR-TB among those under first-line treatment, and therefore can contribute to re-infection. MDR acquisition occurs at an equal rate (σ) for smear-negative and smear-positive disease and detection of newly acquired MDR-TB is unaffected by the use of Xpert MTB/RIF in case finding. For individuals who acquire MDR-TB, we assumed second-line treatment would be initiated at half the rate for cases of MDR-TB newly diagnosed as having TB.

Outcomes were calibrated to expected treatment success rates estimated from past experience in FSU prisons (for base case parameter estimates, see Table 2). Individuals who fail an initial course of treatment for non-MDR TB remain in treatment states for a second first-line treatment course. The addition of streptomycin to this regimen, consistent with WHO category II treatment, was left out for the sake of model tractability. Treatment default as reported in the literature was modeled as remaining on treatment until treatment success, treatment failure or acquisition of MDR-TB, since release from treatment states is dealt with separately in our model. One third of those failing an initial course of treatment were assumed to be successfully treated, one third were assumed to convert to MDR-TB and one third were assumed to transition to chronic TB.

*Costs of screening, diagnosis and treatment*

Taking the health system perspective, costs accrue from baseline health expenditure, case-finding activities, treatment and hospitalization both while incarcerated and after release. Costs associated with case finding activities accrue in two steps. First, the cost of the screening methodology is applied to all inmates in the general population (i.e. all those not currently under TB treatment). This first cost accrues only at the time step of the annual screening activity in strategies including such activities. A second cost—the cost of completing the diagnostic workup—accrues for all those who screen positives (both true positives and false-positives). The full diagnostic workup is composed of MMR, symptom history (assumed to have the same cost as symptom screening) and direct sputum smear microscopy. These activities were not included in the cost of the diagnostic workup if they were part of the screening step. For all inmates who self-refer for care outside the framework of annual screening activities, the cost of a full diagnostic workup accrues. In all cases, the cost of culture and DST is included in the cost of treatment.

The cost of treatment is applied to all those who remain positive for disease after the diagnostic process. In our model, true positives are represented by the transition of individuals from undetected disease to detected disease; therefore the treatment cost associated with true positive disease accrues as a weekly cost for all those in treatment states. False positive results occur in the diagnostic process, at a proportion based on the specificity of the full diagnostic workup (specificity = 0.782) [58]. The cost of a false positive result of the diagnostic process accrues as a total treatment cost, but is not represented by any transition among inmates.

Overtreatment for MDR-TB also occurs in our model at a rate based on the specificity of the diagnostic process leading to transition from first-line treatment to second-line treatment. In strategies including the use of Xpert MTB/RIF, the specificity for MDR-TB was 0.9805 [43]. We made the conservative assumption that the specificity of the MDR diagnosis in the absence of Xpert MTB/RIF was 0.99. Individuals with MDR-TB who have been diagnosed with TB, but await detection of their multidrug resistance, accrue the weekly cost of standard first-line therapy. Individuals under standard first-line therapy who convert to MDR-TB continue to accrue the weekly cost of first-line until their newly acquired multidrug resistance is detected.

*Release*

Costs accrued and QALYs lived after release were projected in a static fashion using simplified age-stratified Markov models (Figure S2). Tuberculosis transmission was not explicitly modeled in these models. Remaining years of life were based on the age distribution of Russian prisoners [17] and, in our base case, WHO life tables from the Russian Federation [18]. For country-specific analyses, WHO life tables and data on baseline health expenditure from Latvia, the Russian Federation and Tajikistan were used [18,19]. Individuals released from latent infection states developed reactivation disease with an annual probability of 0.00141 (based on a lifetime probability of 0.107) [20]. Individuals released with fast-progressing latent infection or with active disease were assumed to undergo treatment immediately upon release. Individuals released with chronic disease had a life expectancy of 5 years.

Future baseline health expenditures and the cost of future treatment were included. Screening/diagnostic costs for active disease outside the prison were not included. Projected years of life were adjusted for quality only during active disease states. Treatment outcomes were assumed to occur with the same probabilities as within the prison. All projected QALYs and costs were discounted at a 3% rate [10]. For country-specific analysis, the inputs to post-release Markov models were matched to available data for each country where possible.

*Cost-effectiveness analysis*

Our transmission model was used in a cost-effectiveness analysis in which costs and QALYs could be directly compared for alternative case finding strategies. Each case finding strategy considered was applied to the transmission model. All QALYs lived by individuals and all health-care costs spent by the health system after entry to the prison transmission model were counted for comparison using both the transmission model and the Markov models simulating release from prison (Figure S3).

*Primary cost analysis*

In early 2010, we conducted interviews with administrators and technicians of the National Reference Laboratory for Tuberculosis; the Global Fund to Fight AIDS, Tuberculosis and Malaria; Caritas, Luxembourg (a non-governmental organization involved in implementing the WHO Directly Observed Therapy Short course strategy in the prison system of Tajikistan); and the Center for Medical and Epidemiological Screening (a local provider of MMR screening in Khudjand, Tajikistan). These interviews were conducted alongside a prevalence survey administered in two prisons in Tajikistan, in which symptom screening, MMR, sputum smear microscopy and culture were performed. Data from our budget for performing this prevalence survey supplemented the data from the interviews. From these sources, we developed estimates of capital, material, labor and indirect costs, depreciating large fixed costs linearly over their expected useful life. These estimates were then aggregated to develop unit costs for screening and diagnostic tests and weekly costs for treatment of non-MDR TB and MDR-TB (Table S7). We compared our estimates to figures reported in the literature from other regions, in order to ensure external validity [59–61]. All costs were converted to 2009 US dollar, using an exchange rate of 2.452 Somoni (Tajikistan) to the US$ and 0.709 Euro to the US dollar, and using actual historical local inflation rates for all past costs [61].

We explicitly modeled the use of the Xpert MTB/RIF, which allows early detection of rifampin resistance. We drew our estimate for the cost of sputum PCR analysis from an adaptation of the estimated ingredient costs reported in a recent large, multi-center in trial cost-effectiveness analysis [14]. Non-tradable costs were projected exponentially according to GDP from itemized point estimates in India, Uganda and South Africa.

*Quality adjustments*

Health states were adjusted for quality of life on the basis of disease severity, side effects of treatment and time spent as inpatient, and were derived from published disease-specific utility weights for tuberculosis health states (Table S8) [15,16]. In order to avoid devaluing the life of prisoners (who should be considered a vulnerable population), quality of life was not adjusted for incarceration status. Quality of life was not adjusted for age, as our primary transmission model is not age-stratified. For susceptible individuals and individuals with latent infection (both MDR and non-MDR strains), quality of life was given a utility weight of 1. Active disease is stratified based on whether treatment has been initiated and on length of time for which individuals would be required to be isolated. Symptomatic disease that remains undetected is given a utility weight of 0.73. Active disease that has been detected and is being treated is stratified by smear-positivity, under the assumption that the requirement of a period of isolation or an extended inpatient stay would result in a meaningful decrement in quality of life. For active disease states that are non-MDR or that are MDR with multidrug resistance yet to be detected––individuals being treated with standard first-line therapy––smear-negative disease is weighted as 0.68 and smear-positive disease as 0.60. Similarly, all individuals with MDR TB on second-line treatment were given a utility weight of 0.60.

*Sensitivity analyses*

We conducted deterministic univariate and two-way sensitivity analyses, as well as a Monte Carlo probabilistic sensitivity analysis. Where possible, parameter ranges for univariate and two-way sensitivity analyses were selected on the basis of 95% confidence intervals for individual parameter estimates. When parameters were estimated from pooled data, confidence intervals were adjusted for unobserved study heterogeneity by reducing the pooled sample size used to compute confidence intervals by 5%. In cases where confidence intervals could not be calculated, the range of reasonable values was estimated subjectively.

We performed a probabilistic sensitivity analysis in which the set of all parameters was sampled 10,000 times. In the absence of information about the covariance of these parameters, we assumed that any two parameters could be either independent or perfectly correlated. All parameters corresponding to probabilities and proportions were sampled from beta distributions. Where available, we obtained the shape parameters of the beta distributions from published studies that listed the number of participants in the study. To combine more studies, we summed the number of participants in each study as well as the number of ‘success’ cases in each study to compute the overall probability of a ‘success’ occurring, and to account for any unobserved heterogeneity in this data we decreased the total number of participants by 5%. When no study was available, we fitted a beta distribution having the base case value as mean and a variance such that the difference between the 97.5 percentile and the 2.5 percentile was approximately equal to the base case +/- 25%. The contact rates for non-MDR and MDR disease (β_d_ and β_m_) were also sampled from the gamma distribution, in order to account for the absence of an upward bound on these parameters.

We simulated all strategies using these 10,000 samples and obtained the cost-effectiveness acceptability curve (CEAC) by computing the net monetary benefit (NMB) for each strategy for each willingness-to-pay (WTP) value between $0 and $15,000 in $500 increments, and determining for each WTP value, how many times out of 10,000 PSA samples the strategy was optimal (had highest NMB).

**References**

46. Resch SC, Salomon JA, Murray M, Weinstein MC (2006) Cost-effectiveness of treating multidrug-resistant tuberculosis. PLoS Med 3: e241. doi:10.1371/journal.pmed.0030241.

47. Murray CJ, Salomon JA (1998) Modeling the impact of global tuberculosis control strategies. Proc Natl Acad Sci USA 95: 13881–13886.

48. Sutherland I, Svandová E, Radhakrishna S (1982) The development of clinical tuberculosis following infection with tubercle bacilli. 1. A theoretical model for the development of clinical tuberculosis following infection, linking from data on the risk of tuberculous infection and the incidence of clinical tuberculosis in the Netherlands. Tubercle 63: 255–268.

49. Vynnycky E, Fine PE (1997) The natural history of tuberculosis: the implications of age-dependent risks of disease and the role of reinfection. Epidemiol Infect 119: 183–201.

50. Behr MA, Warren SA, Salamon H, Hopewell PC, Ponce de Leon A, et al. (1999) Transmission of Mycobacterium tuberculosis from patients smear-negative for acid-fast bacilli. Lancet 353: 444–449.

51. Diel R, Loddenkemper R, Meywald-Walter K, Gottschalk R, Nienhaus A (2009) Comparative performance of tuberculin skin test, QuantiFERON-TB-Gold In Tube assay, and T-Spot.TB test in contact investigations for tuberculosis. Chest 135: 1010–1018. doi:10.1378/chest.08-2048.

52. Tostmann A, Kik SV, Kalisvaart NA, Sebek MM, Verver S, et al. (2008) Tuberculosis transmission by patients with smear-negative pulmonary tuberculosis in a large cohort in the Netherlands. Clin Infect Dis 47: 1135–1142. doi:10.1086/591974.

53. Dye C, Scheele S, Dolin P, Pathania V, Raviglione MC (1999) Consensus statement. Global burden of tuberculosis: estimated incidence, prevalence, and mortality by country. WHO Global Surveillance and Monitoring Project. JAMA 282: 677–686.

54. Drobniewski F, Balabanova Y, Zakamova E, Nikolayevskyy V, Fedorin I (2007) Rates of latent tuberculosis in health care staff in Russia. PLoS Med 4: e55. doi:10.1371/journal.pmed.0040055.

55. Gatner EM, Burkhardt KR (1980) Correlation of the results of X-ray and sputum culture in tuberculosis prevalence surveys. Tubercle 61: 27–31.

56. World Health Organization (2010) Treatment of Tuberculosis: guidelines. World Health Organization.

57. van Cleeff MRA, Kivihya-Ndugga L, Githui W, Nganga L, Odhiambo J, et al. (2003) A comprehensive study of the efficiency of the routine pulmonary tuberculosis diagnostic process in Nairobi. Int J Tuberc Lung Dis 7: 186–189.

58. Dowdy DW, Lourenço MC, Cavalcante SC, Saraceni V, King B, et al. (2008) Impact and cost-effectiveness of culture for diagnosis of tuberculosis in HIV-infected Brazilian adults. PLoS ONE 3: e4057. doi:10.1371/journal.pone.0004057.

59. Mueller DH, Mwenge L, Muyoyeta M, Muvwimi MW, Tembwe R, et al. (2008) Costs and cost-effectiveness of tuberculosis cultures using solid and liquid media in a developing country. Int J Tuberc Lung Dis 12: 1196–1202.

60. van Cleeff M, Kivihya-Ndugga L, Githui W, Ng’ang’a L, Kibuga D, et al. (2005) Cost-effectiveness of polymerase chain reaction versus Ziehl-Neelsen smear microscopy for diagnosis of tuberculosis in Kenya. Int J Tuberc Lung Dis 9: 877–883.

61. Historical Exchange Rates | OANDA (n.d.). Available:http://www.oanda.com/currency/historical-rates/. Accessed 30 August 2011.
